# Murine Norovirus Infection Results in Anti-inflammatory Response Downstream of Amino Acid Depletion in Macrophages

**DOI:** 10.1128/JVI.01134-21

**Published:** 2021-09-27

**Authors:** Michèle Brocard, Jia Lu, Belinda Hall, Khushboo Borah, Carla Moller-Levet, Iliana Georgana, Frederic Sorgeloos, Dany J. V. Beste, Ian G. Goodfellow, Nicolas Locker

**Affiliations:** a Faculty of Health and Medical Sciences, School of Biosciences and Medicine, University of Surrey, Guildford, United Kingdom; b Division of Virology, Department of Pathology, Addenbrooke’s Hospital, University of Cambridge, Cambridge, United Kingdom; Instituto de Biotecnologia/UNAM

**Keywords:** norovirus, stress response, translation

## Abstract

Murine norovirus (MNV) infection results in a late translation shutoff that is proposed to contribute to the attenuated and delayed innate immune response observed both *in vitro* and *in vivo.* Recently, we further demonstrated the activation of the α subunit of eukaryotic initiation factor 2 (eIF2α) kinase GCN2 during MNV infection, which has been previously linked to immunomodulation and resistance to inflammatory signaling during metabolic stress. While viral infection is usually associated with activation of double-stranded RNA (dsRNA) binding pattern recognition receptor PKR, we hypothesized that the establishment of a metabolic stress in infected cells is a proviral event, exploited by MNV to promote replication through weakening the activation of the innate immune response. In this study, we used multi-omics approaches to characterize cellular responses during MNV replication. We demonstrate the activation of pathways related to the integrated stress response, a known driver of anti-inflammatory phenotypes in macrophages. In particular, MNV infection causes an amino acid imbalance that is associated with GCN2 and ATF2 signaling. Importantly, this reprogramming lacks the features of a typical innate immune response, with the ATF/CHOP target GDF15 contributing to the lack of antiviral responses. We propose that MNV-induced metabolic stress supports the establishment of host tolerance to viral replication and propagation.

**IMPORTANCE** During viral infection, host defenses are typically characterized by the secretion of proinflammatory autocrine and paracrine cytokines, potentiation of the interferon (IFN) response, and induction of the antiviral response via activation of JAK and Stat signaling. To avoid these and propagate, viruses have evolved strategies to evade or counteract host sensing. In this study, we demonstrate that murine norovirus controls the antiviral response by activating a metabolic stress response that activates the amino acid response and impairs inflammatory signaling. This highlights novel tools in the viral countermeasures arsenal and demonstrates the importance of the currently poorly understood metabolic reprogramming occurring during viral infections.

## INTRODUCTION

The accumulation of viral double-stranded RNA (dsRNA) replication intermediates or proteins during infection imposes a major stress on the host cell. In response to this stress, infected cells induce several defense mechanisms that promote cell survival and initiate an antiviral program (1–3). This first line of defense culminates in a global inhibition of protein synthesis while allowing the specific translational control instrumental in promoting the NF-κB and type I interferon (IFN)-dependent antiviral innate immune response induced downstream of virus recognition by pattern recognition receptors (pathogen-associated molecular patterns [PAMPs]) (4–8). It involves finely choregraphed events, such as inhibition of the global translation via phosphorylation of α subunit of eukaryotic initiation factor 2 (P-eIF2α) and the assembly of stress granules to sequester the bulk of cytoplasmic mRNAs (9), leading to the specific translation of components of the integrated stress response (ISR) genetic program downstream of P-eIF2α (10, 11) and to the specific recruitment into actively translating polysomes of NF-κB and IRF3/7 target genes transcripts such as tumor necrosis factor (*TNF*), interleukin-6 (*Il-6*), *IfnB1* (12).

The *Caliciviridae* family comprises small nonenveloped positive-strand RNA viruses of medical and veterinary importance (13). Among these, human norovirus (HuNoV) is a major cause of acute gastroenteritis outbreaks worldwide, responsible for more than 200,000 deaths per year, and has a socioeconomic impact estimated at more than $60 billion/year (14, 15). Both HuNoV and murine norovirus (MNV) belong to the *Norovirus* genus and share many characteristics, with MNV benefiting from reverse genetics systems, small animal model, and easy propagation in cell culture (16). Therefore, MNV provides a valuable model for understanding the life cycle of caliciviruses.

MNV replication is sensed by melanoma differentiation-associated protein 5 (MDA5) (17), a member of the RIG-I-like receptors family (RLRs), leading to an IFN response (18). Moreover, while MNV replication is susceptible to IFN pretreatment or activation of the Toll-like receptor (TLR) cascades, these antiviral responses become ineffective after the early phase of infection, suggesting escape from antiviral responses (19, 20). Furthermore, our previous studies have shown that MNV infection regulates translation in several of the following ways: by controlling the activity of multiple eIFs by inducing eIF4E phosphorylation via the mitogen-activated protein kinase (MAPK) pathway and by cleavage of PABP and eIF4G by the viral protease or cellular caspases (19, 21). We and others also recently showed that while MNV impairs global translation, this shutoff is uncoupled from the activation of the eIF2α-dependent stress responses (19, 21–23).

In response to viruses, the ISR is classically linked to the activation of the eIF2α kinases PKR by viral dsRNA replication intermediates or PERK due to the accumulation viral proteins in the endoplasmic reticulum (ER) (10, 24, 25). However, our previous study identified the kinase GCN2 as responsible for MNV-induced phosphorylation of eIF2α. GCN2 is activated by numerous stresses, including amino acid starvation and ribosomal stresses, such as translation elongation defects and ribosome collisions, triggering the ISR downstream of translation inhibition (26–28). Interestingly, several lines of evidence suggest a strong link between metabolic homeostasis, immunotolerance, and immunosuppression correlating with GCN2 activity (29–31). Sensing of metabolic stress also initiates the immediate early response (IER), leading to activation of the transcription factor ATF2 via the MAPK JNK (32). The resulting response to both the IER and ISR, also called the amino acid response (AAR), revolves around the transcriptional activity of the transcription factors ATF4, ATF3, and CHOP leading to a homeostatic response to the identified stress and suppression of NF-κB-dependent inflammation or to apoptosis via the intrinsic apoptosis pathway if this stress cannot be resolved (10). Noticeably, ATF3 had been shown to negatively regulate the transcription of proinflammatory genes, such as *Il-6* and *Il12p40* (30, 33). This, together with the links between GCN2 activity and immunosuppression, and its antiviral roles during Sindbis virus or HIV infection (6, 29–31, 34), puts GCN2 at the nexus between MNV infection, stress responses, and innate immunity.

Herein, we hypothesize that the impaired antiviral response during MNV infection may in part originate from a metabolic stress-activating GCN2-mediated ISR and IER, leading to the suppression of NF-κB-dependent inflammation downstream of MDA5 activation. To test this, we identified an imbalance in amino acid metabolism in MNV-infected cells and characterized the cellular response using genome-wide analysis of the transcriptome, translatome, and proteome in MNV-infected macrophage cell lines. We demonstrated the activation of an MDA5-dependent pathway lacking features of a typical innate immune response and correlating with the activation of an ISR/IER genetic program during MNV infection. We further show the importance of ATF3 upregulation and the ATF/CHOP target Gdf15, a tolerogenic factor and nonsteroidal anti-inflammatory drug-activated gene (NAG-1) (35), in controlling paracrine inflammatory targets, such as Ptgs2 (Cox-2) or Mx1. Altogether, our results highlight a previously undescribed mechanism of control of the antiviral response in macrophages via activation of a metabolic stress response associated with MNV-induced tolerance.

## RESULTS

### MNV induces an amino acid imbalance in infected cells.

**FIG 1:**
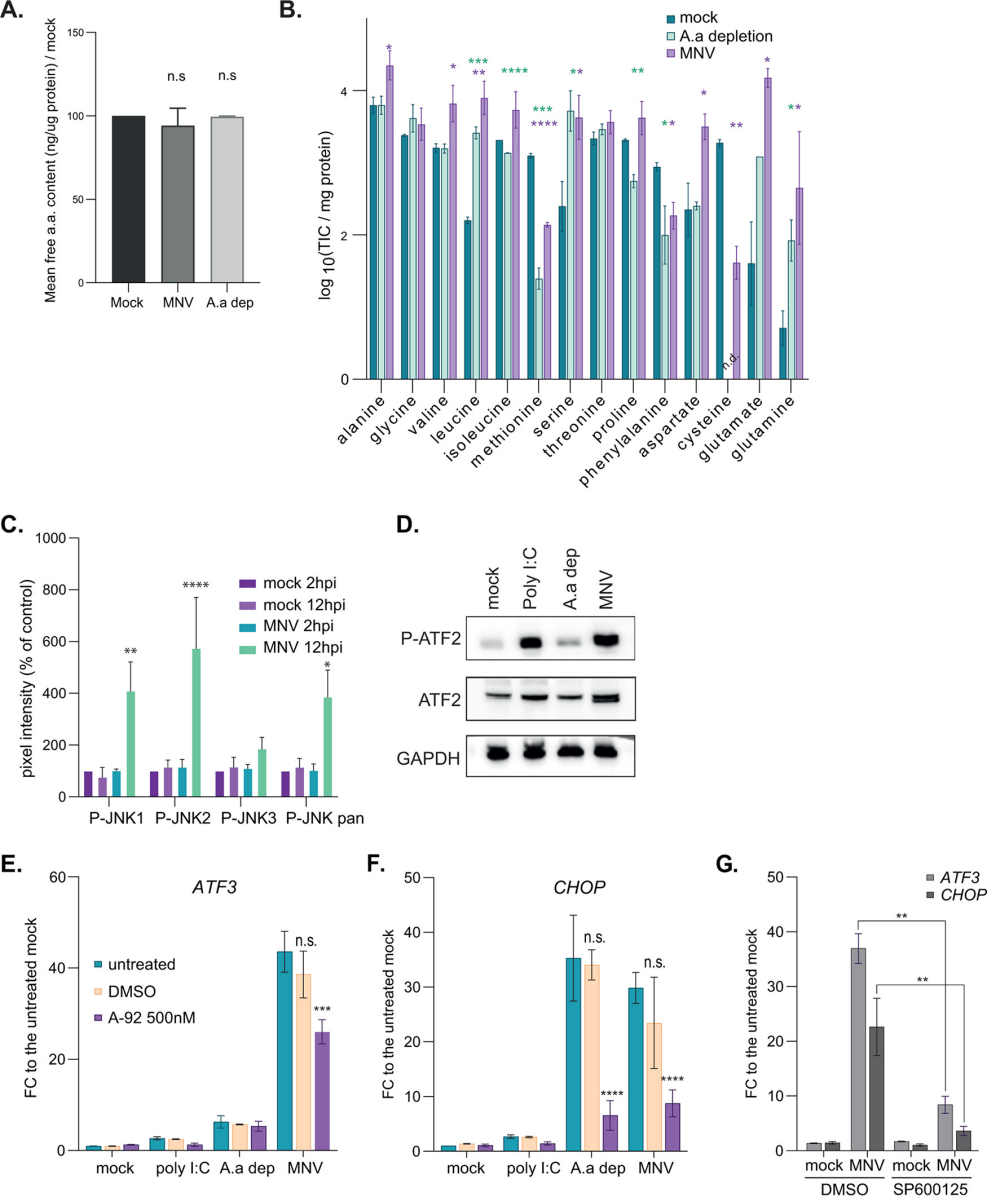
MNV infection is associated with an amino acid (a.a.) depletion phenotype. (A and B) Quantification of free amino acids in RAW264.7 cells mock (dark green) and MNV infected (magenta) for 10 h or incubated in GCM-depleted medium (light green). (A) There were no significant differences between the total concentration of free amino acids quantified using a colorimetric assay. By contrast GC-MS (B) measured significant differences in the pool sizes of individual amino acids (TIC, total ion current). Values are means ± standard errors of the mean (SEM) (*n* = 3) submitted to *t* test pairwise comparison. ****, *P* < 0.0001; ***, *P* < 0.001; **, *P* < 0.01; *, *P *< 0.1. The mass-to-charge ratios for analyzed amino acids are alanine 260, glycine 246, valine 288, norvaline 288, leucine 274, isoleucine 274, methionine 320, serine 390, threonine 404, proline 258, phenylalanine 336, aspartate 418, cysteine 406, glutamate 432, ornithine 417, asparagine 417, lysine 431, glutamine 431, arginine 442, histidine 440, tyrosine 466, tryptophan 375; n.d., not determined. (C) Stress related JNK1/2 are activated during MNV replication in RAW264.7 cells. Bar plot (*n* = 3) of the mean ± standard deviation (SD) of the results of phosphoantibody array assay (*n* = 3) at 2 h p.i. (light) and 12 h p.i. (dark) in mock and cells infected with MNV at an MOI of 10 (green and purple bars, respectively). Statistical analysis results shown above the plots. *, *P *< 0.1; **, *P *< 0.01; ****, *P *< 0.0001. (D) Western blot analysis of the activation of ATF2 in mock cells, cells treated with 20 μg/ml of poly(I·C), amino acid-starved cells and MNV-infected cells at 10 h p.i. (MOI, 10). (E and F) GCN2 is partially responsible for the genetic reprogramming in MNV-infected cells. Bar plots (*n* = 4) of the mean ± SEM of transcript upregulation analysis by qRT-PCR for *ATF3* mRNA (E) and *CHOP* mRNA (F). Experiment performed on total transcripts from RAW264.7 cells treated as in panel B for 10 h with or without 500 nM GCN2 inhibitor A92. Purified RNAs were reverse transcribed using poly(dT) primer and quantitative PCR (qPCR) performed using exon-junction spanning pair of PCR primer. Statistical significance between untreated and treated samples calculated using analysis of variance (ANOVA) two-way multiple comparison tool in GraphPad shown above the bars. ****, *P *< 0.0001; ***, *P *< 0.001; n.s., not significant. (G) Bar plots (*n* = 3) of the mean ± SEM of transcript upregulation analysis by qRT-PCR for *ATF3* mRNA and *CHOP* mRNAs following JNK inhibitions. Experiment performed on total transcripts from RAW264.7 cells treated as in panel E for 10 h with or without 20 μM JNK inhibitor SP600125. Statistical significance between untreated and treated samples calculated using ANOVA two-way multiple comparison tool in GraphPad shown above the bars. ****, *P *< 0.0001; ***, *P *< 0.001; n.s., not significant.

We previously identified a role for GCN2 in inducing eIF2a phosphorylation during MNV infection (23). Given the link between GCN2 activation and the response to amino acid starvation, we assessed amino acid levels in MNV-infected cells by first measuring the total content in free amino acids using a colorimetric enzymatic assay in mock- or MNV-infected RAW264.7 murine macrophage cells at 10 hours postinfection (h p.i.) using cells grown in glycine-cysteine-methionine (GCM)-depleted medium as control. The results showed no significant differences in the total concentration of free amino acids between all conditions, suggesting the absence of global amino acid depletion during infection (Fig. 1A). Next, we quantified the pool sizes of individual amino acids using gas chromatography-mass spectrometry (GC-MS). This analysis revealed significant differences in the pool sizes of free amino acids in MNV-infected cells, interestingly with an overall pattern similar to that observed for cells grown in GCM-depleted medium (Fig. 1B). Specifically, GCM depletion increased glutamine, glutamate, serine, and leucine and reduced methionine, cysteine, and phenylalanine pool size, which was mirrored in MNV-infected cells, suggesting that viral infection induced a similar metabolic stress response as amino acid starvation. Additionally, virally infected cells had increased pool sizes of free valine, isoleucine, proline, and aspartate. Although we cannot make any conclusions about specific changes in metabolic flux from these results, this data confirms that MNV replication induces a metabolic stress response, which disrupts amino acid homeostasis. Beside GCN2 activation, the amino acid response is characterized by the activation of the MAPK pathway, specifically its JNK2 arm, leading to the activation of the AP1 transcription factor ATF2 (32, 36). Our previous analysis in MNV-infected RAW264.7 cells showed the activation of Erk1/2 and p38 activation as part of a classical antiviral response (21). Similar MAPK antibody arrays were used to examine the phosphorylation of JNK isoform at 2 and 12 h p.i. in mock- and MNV1-infected RAW264.7 cells. These data, summarized in Fig. 1C, show an increase in phosphorylated JNK2 at 12 h p.i. and, to a lesser extent, in JNK1 in MNV-infected cells. These results further support the sensing of a metabolic imbalance and activation of the AAR, encompassing the observed activation of GCN2 on one hand and the activation of the JNK pathway on the other. Next, we used immunoblotting to examine the phosphorylation status of ATF2 at Thr71 in MNV-infected cells (Fig. 1D). We then confirmed by reverse transcription-quantitative PCR (qRT-PCR) the upregulation of the ISR and IER response genes *ATF3* and *CHOP/Ddit3* in MNV-infected cells (Fig. 1E and F), which can be partially reverted by the GCN2 inhibitor A92, at a concentration known to inhibit the GCN2-dependent phosphorylation of eIF2α in MNV-infected cells (23). Similarly, the contribution of JNK activation was assayed using the selective inhibitor SP6100125. Pharmacological inhibition of JNK resulted in impaired upregulation of *ATF3* and *CHOP/Ddit3* in MNV-infected cells (Fig. 1G). Altogether, these results suggest the activation of AAR signaling downstream of an amino acid imbalance, culminating in the expression of its associated genetic program during MNV infection.

### Genetic reprogramming analysis reveals an AAR response in MNV-infected cells.

**FIG 2:**
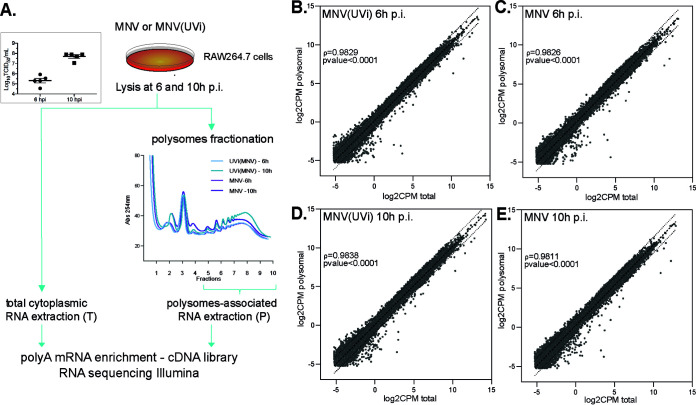
Multivariate omics analysis of MNV-infected cells by RNA sequencing. Comprehensive genome-wide RNA sequencing analysis comparing the changes of the landscape of cytoplasmic transcripts available for translation (total fraction) to the corresponding changes of the landscape of transcripts recruited into polysomes (polysomal fraction), their kinetic variation from 6 to 10 h p.i., in murine macrophage cell line RAW264.7 infected with the replicative MNV (MOI, 10) or the nonreplicative UV-inactivated MNV, MNV(UVi). (A) Diagram of the experimental design for the translatomic analysis, from infection to RNA sequencing, also showing in insert the similar efficiencies of MNV infection across samples and replicates addressed by measure of the viral titer by TCID_50_ (logarithmic scale) at 6 and 10 h p.i. (B to E) Scatterplots of the RNA sequencing results plotted as the average log_2_CPM in polysomal (*y* axis) versus total fraction (*x* axis) for each condition, MNV(UVi) (B and D) or MNV (C and E) at 6 (B and C) or 10 h p.i. (D and E). The log_2_CPM of the host data set genes were obtained by library size normalization of the raw results and filtering out of the low read genes (CPM < 8). Correlation analysis using the ‘simple linear regression’ function from GraphPad showing the strong relationship (Spearman correlation coefficient *ρ* > 0.98; pval < 0.0001) between the total and polysomal fractions. Dotted lines, linear regression and 95% confidence intervals.

The homeostatic response AAR is responsible for starvation-induced survival and suppresses any further inflammation via the ATF4 and ATF2 response gene *ATF3* (37, 38), partly by inhibiting the upregulation of NF-κB target genes *Il-6* and *Il12p40* (30, 33). To understand the global impact of this amino acid imbalance on the host antiviral response to MNV infection, we performed a genome-wide analysis of the transcriptome and translatome. Transcriptome (RNA-Seq) analysis of the cytoplasmic poly(A)-tailed transcripts (total fraction) and poly(A)-tailed transcripts associated with polysomes (polysomal fraction) was carried out at 6 and 10 h p.i., corresponding to time points prior to and during the MNV-induced phosphorylation of eIF2α by GCN2 (23) in RAW264.7 cells infected at a multiplicity of infection (MOI) of 10 with matching controls using UV-inactivated virus—MNV(UVi) (Fig. 2A). Transcripts associated with polysomes were isolated after ultracentrifugation through sucrose gradients and pooling of fractions 5 to 10 (see Fig. S1A to C in the supplemental material). Three independent replicates were used, with similar viral replication efficacy across replicates as analyzed by 50% tissue culture infective dose (TCID_50_) (Fig. 2A, insert). The extracted RNA samples were evaluated for concentration and quality with a Bioanalyzer before analysis by RNA-Seq. All sample reads were aligned to murine genome assembly GRCm38.p5 using Hisat2 and MNV1-CW1 genomic RNA (GenBank accession number DQ285629) using Bowtie 2 (Fig. S1D and E). This revealed that 98.2% ± 0.2% and 97.4% ± 0.06% of reads aligned to the host genome in total and polysomal fractions, respectively, at 6 h p.i., and this decreased to 69.9% ± 1.7% and 71.4% ± 1.4% at 10 h p.i., reflecting increased viral RNA synthesis and translation (Fig. S1E). Biorthogonal plotting of the averages of normalized reads (log_2_CPM) of the three replicates for the different conditions showed a striking relationship between polysomal and total cytoplasmic fractions using the nonparametric Spearman correlation test (ρ > 0.98; *P* value [pval] < 0.0001 across all of the conditions), supporting a global transcript recruitment into the polysomes as a function of its cytoplasmic availability even in MNV-infected samples at 10 h p.i. (Fig. 2B to 2E). Further filtration of the data sets by gene feature annotation led to an enrichment for the protein coding transcripts of 85.9% (Fig. S1F). Correlation analysis showed strong similitude (Pearson correlation coefficient *r* > 0.98) with clear hierarchical clustering following the parameters infection, time, fractions, and no predominant batch effect (Fig. S1G). As differential expression analysis requires compositional normalization and considering the vast amount of MNV RNA in the 10 h p.i. samples, we created sub-data sets normalized by library size omitting the MNV RNAs (host data set). Three-dimensional (3D) principal-component analysis (PCA) of the complete data set (MNV + host genes) showed an expected clustering of the MNV-infected samples (PC1) over other parameters. Similar analysis on the host data set while showing a more complex relationship between samples nevertheless highlighted a clear clustering of the MNV samples at 10 h p.i. following mainly the fraction parameter (PC1) and a resultant of infection, time, and replicate parameters (PC2 and PC3) (Fig. S1H and I).

**FIG 3:**
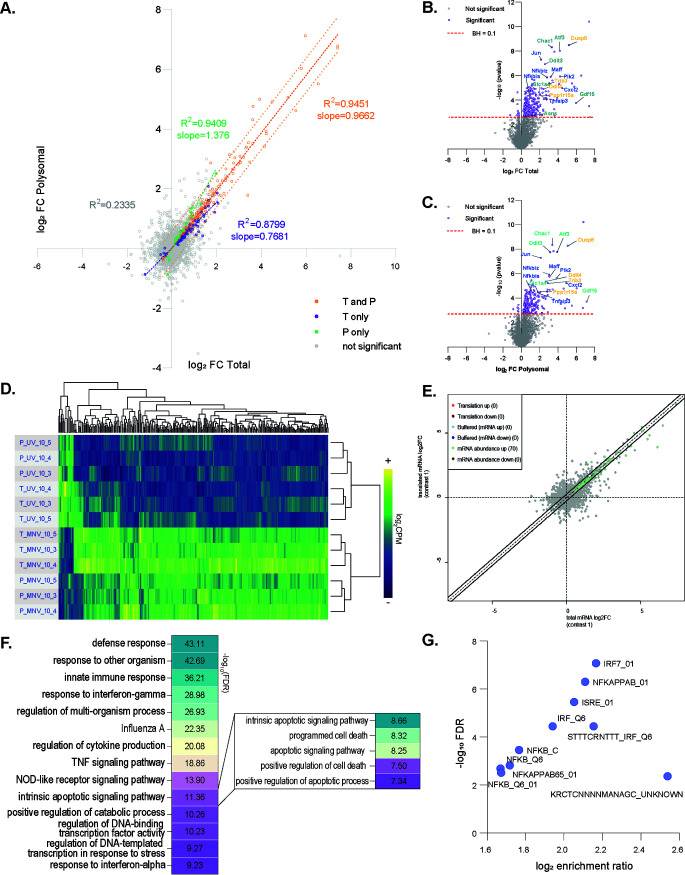
MNV infection induces an attenuated innate immune response as seen by RNA sequencing of both total and polysomal fractions in RAW264.7 cells at 10 h p.i. Differential analysis performed on RNA sequencing data showed a genetic reprogramming induced by MNV replication in RAW264.7 cells lacking both NF-κB and JAK/STAT response. (A) Scatterplot of the results of the differential expression analysis comparing MNV-infected cells to MNV(UVi)-infected cells at 10 h p.i. in the total (*x* axis) versus the polysomal fraction (*y* axis), showing mainly a subset of MNV-specific upregulated genes with similar behavior in both fractions. The differential expression analysis was performed on all samples at 10 h p.i. using the multivariate tool GLM EdgeR, and gene level changes were considered significant when associated with a BHpval of <0.1. Results expressed as log_2_(FC MNV versus MNV(UVi)) of the complete gene data set were used for the biorthogonal projection and subdivided into four groups as follows: genes significant in both total and polysomal fractions (orange dots), genes significant in total fraction only (purple dots), genes significant in polysomal fraction only (green dots), and genes displaying no changes (gray dots). Correlation analysis using the Pearson method showed a high degree of correlation for the three significant groups as indicated on the plot. Linear regression (full orange line) and prediction bands at 95% confidence of significance for the “significant in both fractions” genes (dotted orange lines) seemed to encompass most of the genes significant in one fraction, only reflecting the stringency of the significance cutoff and the likeliness of changes in both fractions for all of the significant genes. (B and C) Volcano plots of the log_2_FC (*x* axis) versus the corresponding −log_10_(pval) (*y* axis) for each gene of the total (B) or the polysomal data set (C) from the differential expression analysis of MNV- versus MNV(UVi)-infected cells at 10 h p.i. showing the high degree of significance (total pval < 0.0025; polysomal pval < 0.002) resulting from the BHpval < 0.1 threshold. Threshold of significance BHpval < 0.1 (red line), significant genes (purple dots), nonsignificant genes (gray dot). Classical target genes downstream of the ISR via ATF4 (yellow) and downstream the IER via ATF2 (blue) and via both ATF4 and ATF2 in response to amino acid starvation (green). (D) Heatmap of comparison of the significant differentially expressed genes in MNV-infected cells displaying their log_2_CPM values in each sample showing a strong clustering following the parameter infection and fraction but not the set of biological replicates. Log_2_CPM values of significant genes for all samples were centered and scaled and subjected to correlation analysis using the Pearson method and complete linkage hierarchical clustering. (E) Scatterplot of the translational control analysis results showing an absence of translational regulation in MNV-infected cells, translated mRNA (*y* axis) versus total mRNA (*x* axis). (F) Clustered results of the gene ontology analysis showing enrichment of MNV-induced upregulated genes in antiviral innate immune response terms and the intrinsic apoptotic pathway. Insert showing the different terms associated within the “intrinsic apoptotic pathway” cluster. The gene annotation analysis was performed in Biological Process and KEGG pathway terms using the ClueGO plug-in on the Cytoscape platform for all genes significantly regulated at 10 h p.i. (total and polysomal data set) and ordered by decreasing corresponding −log_10_(FDR). (G) Scatterplot of the transcriptional network analysis results for the MNV-induced upregulated genes at 10 h p.i. showing enrichment for the IRF, NF-κB, and IFN-response gene pathway. Enrichments were calculated on the transcription factor target database MSigDB using the overrepresentation analysis method and plot created by biorthogonal projection of the log_2_ enrichment ratio (*x* axis) versus the −log_10_(FDR) (*y* axis).

Initial differential expression analysis in total and polysomal fractions between MNV and MNV(UVi) conditions at 6 and 10 h p.i. highlighted a strong overlap of behavior for the majority of genes as suggested by their projection membership into the 95% interval of confidence of significance in both conditions (see Fig. S2A and B in the supplemental material). This overlap likely reflects the physiological changes that occur to the cells during the experiment rather than a direct response to infection *per se*. The heatmap of differentially expressed genes between 6 and 10 h p.i. in MNV-infected samples highlights a lack of clear difference between MNV and MNV(UVi)-infected samples with a confused clustering following infection, time, and fraction parameters (Fig. S2C), suggesting weakening of the statistical analysis due to the introduction of too many variability parameters between isogenic samples and increasing the noisiness of the results. We therefore performed the more robust comparison of the samples prepared temporally in a parallel manner, MNV versus MNV(UVi), at 6 and 10 h p.i. While we did not detect any significant differentially expressed genes at 6 h p.i. between MNV- and MNV(UVi)-infected cells (Fig. S2D), we identified 265 differentially expressed genes in the total fraction and 229 differentially expressed genes in the polysomal fraction (Fig. 3A) at 10 h p.i., all of which displayed a *P* value inferior to 0.0025 for BHpval < 0.1 (Fig. 3B and C). Validation of those results by RT-qPCR showed a significant correlation (Spearman ρ = 0.9564; pval < 0.0001) for the assayed genes (Fig. S2E). While 67 genes showed a significant differential expression only in the total fraction and 31 genes only in the polysomal fraction, the majority fall into a 95% prediction band of coregulation, which seems to confirm the recruitment of transcripts to the polysomes fraction according to their abundance. A hierarchical clustered heatmap of the Log_2_CPM of all the significant differentially expressed genes across the replicates confirmed the strong influence of the infection parameter as the source for differences of expression (Fig. 3D). Moreover, translational efficiency analysis performed with the R package anota2seq (39) highlighted a lack of general translational control in MNV-infected cells at 10 h p.i. (Fig. 3E), leaving only the partial translation shutoff previously described.

Interestingly, most of the identified differentially expressed genes are upregulated (281 out of 296), which is in stark contrast with previous published transcriptomic data of MNV-infected cells (40, 41) and could reflect the bias introduced by the comparison of temporally unmatched conditions in previous studies (i.e., MNV 12 h p.i. versus mock or MNV 0 h p.i.) and low level of infection. Direct comparison showed 31 genes upregulated in all 3 studies in the total fraction, which are quite likely to be the core signature of MNV infection and illustrate the absence of downregulated genes, likely to be linked to physiological changes over time in the previous studies (see Fig. S3A in the supplemental material). Functional annotation analysis and group clustering using Cytoscape (42) (Fig. 3F; see also Fig. S3B) showed enrichment of the upregulated genes for an apparent classical antiviral response with “defense response to organism” (−log_10_(FDR) = 43.11), “response to other organisms” (−log_10_(FDR) = 42.69), “innate immune response” (−log_10_(FDR) = 36.21), “response to cytokine” (−log_10_(FDR) = 34.87), “response to interferon gamma” (−log_10_(FDR) = 28.98), “influenza A” (−log_10_(FDR) = 22.35), or “TNF signaling” (−log_10_(FDR) = 18.86). These results support the 2 previous transcriptomic analyses showing the activation of MAPK kinase and pathways downstream of MDA5 activation during MNV replication and confirmed by transcription target factor analysis and enrichment for the interferon regulatory factors (IRF) and interferon stimulated response element (ISRE) target genes (Fig. 3G). In addition, MNV-induced differentially expressed genes are enriched for “positive regulation of catabolic process” (−log_10_(FDR) = 10.26), which matches the observed MNV induction of autophagy (43). Genes upregulated in MNV-infected cells also showed a strong enrichment for “intrinsic apoptotic signaling pathway” (−log_10_(FDR) = 11.36), encompassing different cell death-related GO terms but only mentioning the intrinsic aspect (−log_10_(FDR) = 8.67) (Fig. 3F, insert), fitting the known activation of the intrinsic apoptotic pathway in MNV-infected cells (44). The 15 downregulated differentially expressed genes did not show any enrichment using the same setting; however, a more relaxed metascape-driven analysis highlighted an enrichment in “positive regulation of cell cycle” (i.e., *E2F2* and *E2F7*, −log_10_(pval) = 4.21), also fitting a previous publication on the effect of MNV on the mitotic activity of the infected cells, especially the G_1_ to S phase transition (45).

Interestingly, the genes enriched in “NOD-like receptor pathway” are the IER target genes downstream of ATF2 activation (*Tnf*, *NFκBia*, *Tnfaip3*, *Cxcl2* [46]). Moreover, those enriched in the “intrinsic apoptotic signaling pathway” (−log_10_(FDR) = 11.36) and “regulation of DNA-templated transcription in response to stress” (−log_10_(FDR) = 9.27) are known ISR-induced genes, such as *ATF4*, *ATF3*, *Ddit3* (CHOP), *Chac1*, *Ppp1r15a* (GADD34), *Trib3*, *Bcl2l11* (Bim), *Dusp2*, and *Dusp8*, and belong to the ATF4-CHOP apoptotic pathway described downstream of the ISR (10, 11, 47). Importantly, the upregulation of *Ddit3* expression (Total_log_2_FC = 2.57) in the absence of *Hspa5* upregulation (Total_log_2_FC = 0.15) is classically linked to nutrient starvation in the absence of unfolding protein response stimulation (48), which also points to the identified metabolic stress. Furthermore, the upregulation of *Ddit3* transcripts confirmed the activation of both the ISR and ATF2 pathways concomitantly, as both are necessary for its transcriptional activation (49). These results confirm the induction of the AAR genetic program in MNV-infected cells downstream of both the ISR and the IER.

In contrast, a comparison with the previously identified lipopolysaccharide (LPS)-induced genes in RAW264.7 cells (12) showed a strong defect in the cytokine induction required for autocrine/paracrine establishment of an antiviral environment downstream of NOD-like receptor (NLR) and TLR activation (4, 7, 8). Specifically, we observed no upregulation of proinflammatory chemokines and cytokines coding genes such as *Il-6*, *Il-18*, *Il-1α*, *Il-1β*, *Il-23*, *Ccl5*, or *Il-12p40* (7) (see Fig. S4A in the supplemental material), known NF-κB target genes, and *Csf2* and *Ptgs2* linked to Stat5 transcriptional activity in bystander cells (50). Transcriptional network analysis of these genes further highlights the strong enrichment in NF-κB binding motifs in their promoters (Fig. S4B). Moreover, the expected programmed cell death downstream of NLR and TLR via the extrinsic apoptotic pathway, necroptosis, and pyroptosis (51), and depending on NF-κB induction of *Fas* or *Tnfrsf10b*, also seems to be absent in MNV-infected cells (Fig. S4C). Altogether, these results reflect the known activation of MDA5 in MNV-infected cells but indicate an inappropriately attenuated NF-κB activity downstream of MDA5, further suggesting a suppressive mechanism correlating with the induction of the anti-inflammatory AAR program.

### Antiviral and anti-inflammatory pathways are both activated in MNV-infected macrophages.

**FIG 4:**
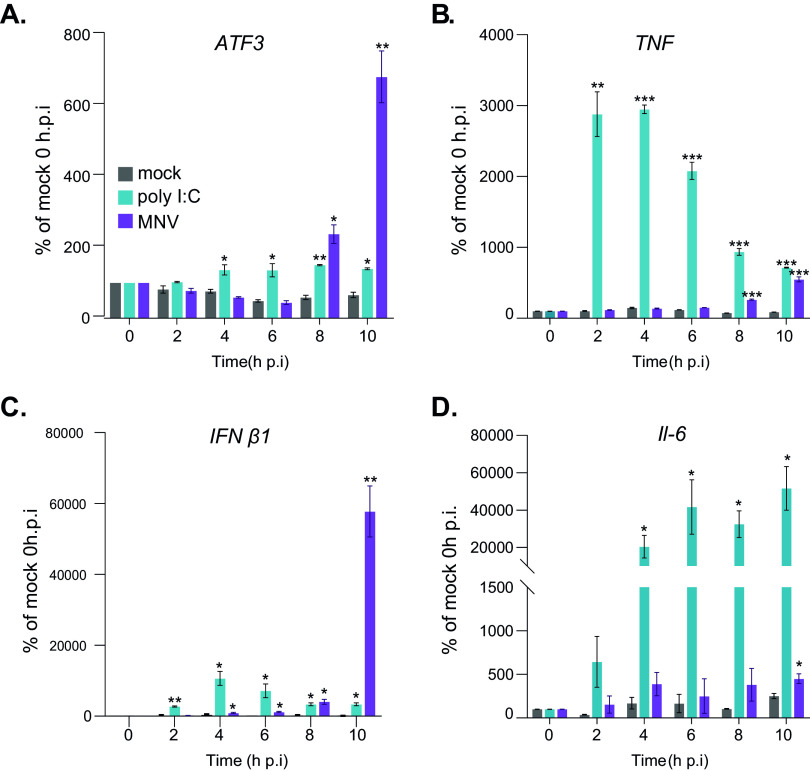
ATF3 response is not secondary to MDA5 response during MNV infection. qRT-PCR analysis of a time course experiment of RAW264.7 cells mock (gray), poly(I·C) treated (cyan), or infected with MNV (purple) at an MOI of 10 for 10 h. Representative results (*n* = 3) are presented as mean ± SD showing the kinetic of regulation of *ATF3* mRNA (A), *TNF* mRNA (B), *Ifnβ1* mRNA (C), and *Il-6* mRNA (D). Statistical analysis results are shown above the plots. *, *P* < 0.1; **, *P* < 0.01; ***, *P *< 0.001.

We, therefore, addressed the kinetics of upregulation of *ATF3*, *TNF*, *Il-6*, and *IFNβ1* gene expression in MNV-infected cells compared with that of cells activated by the addition of poly(I·C) to the medium using by qRT-PCR (52). *ATF3* expression is strongly activated between 6 and 8 h p.i. prior or concomitantly to the upregulation of *IFNβ1* and outstandingly in the absence of priming *TNF* upregulation, which is only seen for the poly(I·C)-treated cells (Fig. 4A to D). This confirms that activation of a proinflammatory signaling pathway by cytokine-mediated amplification loops is missing in MNV-infected cells prior to the modulatory ATF3 response. Rather, MNV replication seems to induce ATF3-dependent anti-inflammatory signaling at the same time as the MDA5-mediated cellular innate immune response.

**FIG 5:**
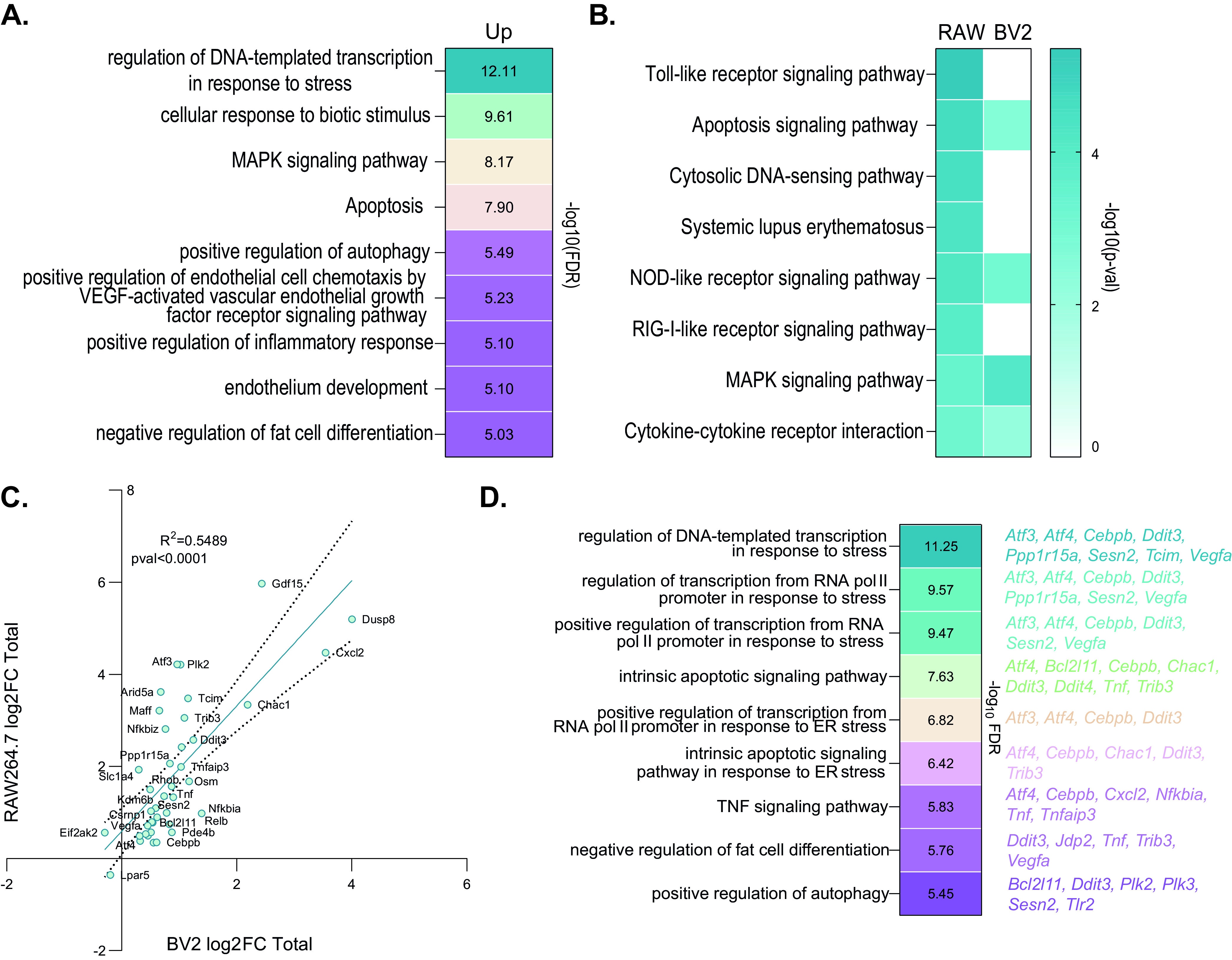
Comparison of transcriptional reprogramming in BV2 and RAW264.7 cells confirms activation of the AAR independently from the NF-κB proinflammatory response during MNV replication. Analysis of the differentially expressed genes in RAW264.7 and BV2 cells lacking the IFN response downstream of MDA5 showed a strong enrichment for the immediate early response combined with the ISR genetic program of expression, including the intrinsic apoptotic pathway but no proinflammatory response or inflammatory-related cell death pathway. (A) Clustered results of the gene ontology analysis showing enrichment of MNV-induced upregulated genes in response to stress terms and apoptosis pathway. The gene annotation analysis was performed in Biological Process and KEGG pathway terms using the ClueGO plug-in on the Cytoscape platform for all genes significantly regulated at 10 h p.i. (total and polysomal data set) and ordered by decreasing corresponding −log_10_(FDR). (B) Comparison of the KEGG and Panther pathways between RAW264.7 and BV2 MNV-infected cells showing absence of proper response downstream of cytoplasmic activation of RIG-I/MDA5 pathways in MNV-infected BV2 cells. The gene annotation analysis was performed on KEGG and Panther pathways and ordered by decreasing corresponding −log_10_(FDR). Blue square, significant enrichment; white square, absence of enrichment. (C) Scatterplots of all the genes significant in both RAW264.7 and BV2 cell lines in either total fraction, plotted using their log_2_FC from the differential expression analysis and showing a positive correlation in both fractions using the Pearson method for pair correlation (total *r *= 0.7409, pval < 0.0001; polysomal *r = *0.7366, pval < 0.0001, 95% infective concentration [IC_95_]). (D) Clustered results of the gene ontology analysis showing enrichment of MNV-induced upregulated genes in response to stress terms and intrinsic apoptotic pathway. (Right) Genes associated with the corresponding cluster. Each cluster contains transcription factors and target genes related to the ISR and the immediate early response with the exception of the cluster “TNF signaling pathway” containing only immediate early response TNF-related genes, such as *TNF*, *Cxcl2*, the inhibitors of NF-κB response *NFκBia* and *Tnfaip3*, and no proinflammatory target genes. The gene annotation analysis was performed in Biological Process and KEGG pathway terms using the ClueGO plug-in on the Cytoscape platform for all genes significantly regulated at 10 h p.i. (total and polysomal data set) and ordered by decreasing corresponding −log_10_(FDR).

To confirm the activation of ATF3-dependent anti-inflammatory signaling independently from the IFN response, we explore the upregulation of the AAR program in BV2 cells, another cell line supporting MNV replication, at 10 h p.i. in mock-, MNV(UVi)-, and MNV-infected cells using the same RNA-Seq pipeline as for RAW264.7. Exploratory and correlation analyses revealed very similar results for both cell lines, including recruitment of given transcripts into the polysomes mostly proportional to their abundance (see Fig. S5A to G in the supplemental material). Differential expression analysis identified 46 upregulated and 19 downregulated genes in the total fraction and 69 upregulated and 25 downregulated genes in the polysomal fraction (see Fig. S6A in the supplemental material). Forty-one genes are significantly differentially expressed in both fractions with a strong linear correlation (Pearson *r *= 0.94; pval < 0.0001), 24 genes are significantly differentially expressed in total fraction only (*r *= 0.31; pval < 0.0001), and 53 genes are significantly differentially expressed in polysomal fraction only (*r *= 0.67; pval < 0.0001). Most of those genes fall in the 95% prediction band of coregulation, and further analysis showed no significant translational control or buffering in MNV-infected BV2 (Fig. S6B and C). Validation of those results by qRT-PCR showed a significant correlation (Fig. S6C). Analysis of the transcription factor network showed the predominance of the ATF family and CHOP transcriptional factors with a complete absence of enrichment for the IRF, ISRE, and NF-κB target genes (Fig. S6D). GO terms analysis of the upregulated genes with Cytoscape showed an enrichment in response to stress with transcriptional activity “regulation of transcription from RNA polymerase II promoter in response to stress” (group −log_10_(FDR) = 12.11), “cellular response to biotic stimulus” (group −log_10_(FDR) = 9.61), activation of the MAPK pathway “MAPK signaling pathway” (group −log_10_(FDR) = 8.17), and stress-induced cell death “apoptosis” (group −log_10_(FDR) = 7.90) (Fig. 5A). The 15 downregulated genes showed no enrichment using Cytoscape. Comparison of the pathways associated with the upregulated genes in MNV-infected RAW264.7 and BV2 cells highlighted the absence of IFN response downstream of MDA5 in BV2 cells (see “Toll-like receptor pathway” and “RIG-I-like receptor signaling pathway”) and emphasized primarily the activation of ATF2 and ATF4 pathways in relation to cell death (Fig. 5B). Genes enriched in “NOD-like receptor pathway” are also an immediate response to stress-activated genes (*Tnf*, *NFκBia*, *Tnfaip3*, *Cxcl2* [46]). In total, 40 genes are differentially expressed in both RAW264.7 and BV2 cells regardless of the fractions: 38 genes upregulated in both cell lines, 1 gene downregulated (*Lpar5*) in both cell lines, and one gene downregulated in BV2 and upregulated in RAW264.7 (an IFN response gene [*Eif2ak2*]) (Fig. 5C). Genome ontology analysis showed significant enrichments and highlighted a response to stress with transcriptional activity “regulation of DNA-templated transcription in response to stress” (−log_10_(FDR) = 11.25) and the ATF4-CHOP-related cell death “Intrinsic apoptotic pathway” (−log_10_(FDR) = 7.63) supporting the activation of ATF4-CHOP apoptotic pathway independently of IRF7 (Fig. 5D). We also observed the same upregulation of the ATF3-Chac1 response in both cell lines when infected with MNV, which suggests the activation of this pathway independently of *Ifnβ1* upregulation of expression. Similarly, the upregulation of *Ddit3* expression in the absence of *Hspa5* upregulation (total log_2_FC = 2.57 in RAW264.7 and total log_2_FC = 1.24 in BV2 cells) associated with nutrient starvation in the absence of UPR stimulation points to an ISR-activating stress different from the ER stress (48). These results further confirmed the upregulation of an immunomodulatory set of genes linked to the ISR pathway and ATF2 activation independently of the MDA5-induced antiviral response during MNV infection.

**FIG 6:**
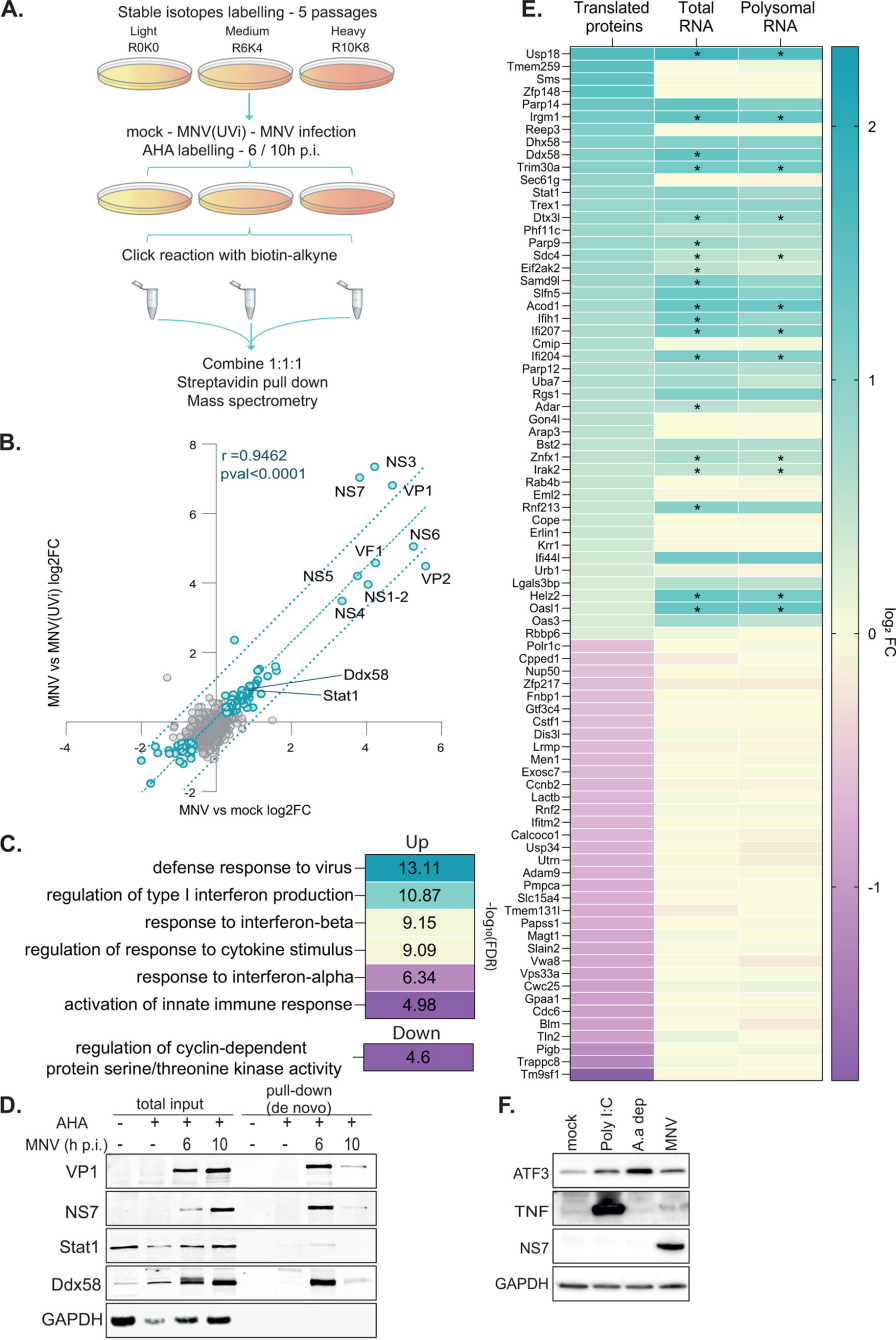
Validation of the results of the translatomic data analysis by mass spectrometry analysis of the nascent proteome in RAW264.7 cells. Genome-wide analysis of the nascent proteome by biorthogonal labeling of the nascent protein and mass spectrometry allowing an extensive validation of the active translatome identified by RNA sequencing. (A) Diagram of the experimental design of the biorthogonal dual labeling proteomics assay as described in reference 53 from SILAC labeling to mass spectrometry. (B) Scatterplot of the results of the differential expression analysis in the contrasts MNV-/mock-infected cells (*x* axis) versus MNV-/MNV(UVi)-infected cells (*y* axis) at 10 h p.i. showing the enrichment for viral structural and nonstructural proteins as well as a subset of MNV-induced differentially expressed host proteins with similar behavior in both contrasts as shown by the correlation analysis. The differential expression analysis was performed on all samples at 10 h p.i. using a pairwise ratio, and results expressed as log_2_(FC) of the complete gene data set were used for the biorthogonal projection, significantly regulated protein (green dots) and not significantly regulated protein (gray dots). Correlation analysis using the Pearson method showed a high degree of correlation between the two contrasts (*R^2^* > 0.89; pval < 0.0001). Dotted blue line, linear regression and IC_95_ of significance. (C) Clustered results of the gene ontology analysis showing enrichment of the upregulated host proteins in antiviral innate immune response terms and enrichment of the downregulated host proteins in regulation of the cell cycle. The gene annotation analysis was performed in Biological Process and KEGG pathway terms using the ClueGO plug-in on the Cytoscape platform for all genes significantly regulated at 10 h p.i. and ordered by decreasing corresponding −log_10_(FDR). (D) Validation of the proteomics analysis setup and results. Representative Western blot against viral proteins and two host proteins upregulated in the proteomics at 10 h p.i. on the inputs (total input) and pulldown fractions of AHA-labeled and lysed RAW264.7 cells at 6 and 10 h p.i. followed by click reaction and streptavidin pulldown, using AHA-unlabeled cells as a negative control. (E) Comparison of the proteomic and translatomic results in RAW264.7, showing both differential expression and differential protein stability in MNV-infected cells at 10 h p.i., with a decrease of stability for all of the proteins identified as downregulated in the proteomic analysis. Heatmap of comparison of the log_2_(FC) of the genes identified by proteomic in MNV versus MNV(UVi) (translated proteins) against their corresponding log_2_(FC) in total (total RNA) and polysomal fraction (polysomal RNA) generated from the translatomic data. Stars represent significant differential expression as described in Fig. 1A. (F) Validation by Western blotting of some of the upregulated genes in MNV-infected RAW264.7 cells identified by RNA sequencing but absent from the proteomic data set. Mock and MNV-infected cells lysates were run on SDS-Page alongside poly(I·C)-treated cells at 20 μg/ml and amino acid-starved cells for 10 h as positive control for the upregulation of TNF and ATF3 respectively.

Next, we confirmed the ability of infected cells to actively translate this transcriptional reprogramming. We measured temporal changes in the nascent proteome by biorthogonal noncanonical amino acid tagging (53), followed by mass spectrometry in cells labeled with the amino acid analogue l-azidohomoalanine (AHA) at 6 and 10 h p.i., comparing mock-, MNV(UVi)-, and MNV-infected RAW264.7 cells (Fig. 6A). This analysis identified 2,661 host and 6 viral proteins at 6 h p.i. and 3,396 host and 9 viral proteins at 10 h p.i. Pairwise ratio comparison identified 56 proteins differentially translated at 6 h p.i. (see Fig. S7 in the supplemental material) and 89 proteins at 10 h p.i. when comparing MNV versus mock and MNV versus MNV(UVi) (Fig. 6B). No specific enrichment was found for the 19 upregulated proteins at 6 h p.i., but the 37 downregulated proteins were enriched for “positive regulation of mitotic nuclear division” (3 genes, −log_10_(FDR) = 3.67). Functional annotation analysis of the results at 10 h p.i. (Fig. 6C) revealed an enrichment of the 52 upregulated genes in “defense response to virus” (−log_10_(FDR) = 13.11), with several IFN type I related GO terms showing the solid transactivation of this response as follows: “regulation of type I interferon production,” −log_10_(FDR) = 10.87; “response to interferon-beta,” −log_10_(FDR) = 9.15; and “response to interferon-alpha,” −log_10_(FDR) = 6.34. However, there was no enrichment in the TNF or NF-κB pathways. The 37 downregulated proteins were enriched in “regulation of cyclin-dependent protein serine/threonine kinase activity” (−log_10_(FDR) = 4.6), which could also contribute to the observed effect of MNV on the cell cycle (45) (Fig. 6C). We confirmed the upregulation of viral proteins and host proteins by immunoblotting analysis of total and pulldown fractions of AHA-labeled and lysed RAW264.7 cells at 6 and 10 h p.i. followed by click reaction and streptavidin pulldown (Fig. 6D). Comparative analysis with the RNA-Seq data sets (Fig. 6E, 83 genes) showed a positive correlation of behavior for a subset of upregulated genes (Pearson *r* = 0.5943; pval < 0.005) belonging to the IFN response, such as *Usp18*, *Irgm1*, or *Ddx58* (RIGI), confirming the translation of a specific transcriptional program in MNV-infected cells, albeit reduced as shown previously (23), and an autocrine IFN response. A subset of genes was significantly translationally upregulated but showed no significant differential expression in the RNA-Seq analysis, reflecting differences of stringency between both methods (i.e., *Parp14* and *Dhx58*) or specific changes in protein stability during infection (i.e., *Tmem259*). Downregulated proteins showed no correlation with the RNA-Seq analysis (i.e., *Tm9sf1* and *Trappc8*), suggesting a decrease in stability for those proteins. The absence of obvious targets of the ATF4/ATF3 pathway among the proteins identified by stable-isotope labeling by amino acids in cell culture (SILAC) prompted us to use immunoblotting to assess the upregulation of ATF3 and inhibition of TNF expression during infection when compared to poly(I·C) treatment (Fig. 6F). These results support the activation of an immunomodulatory pathway and an attenuated NF-κB response, together, rather than downstream of, during MNV infection.

### Secreted GDF15 contributes to immunosuppression during MNV infection.

**FIG 7:**
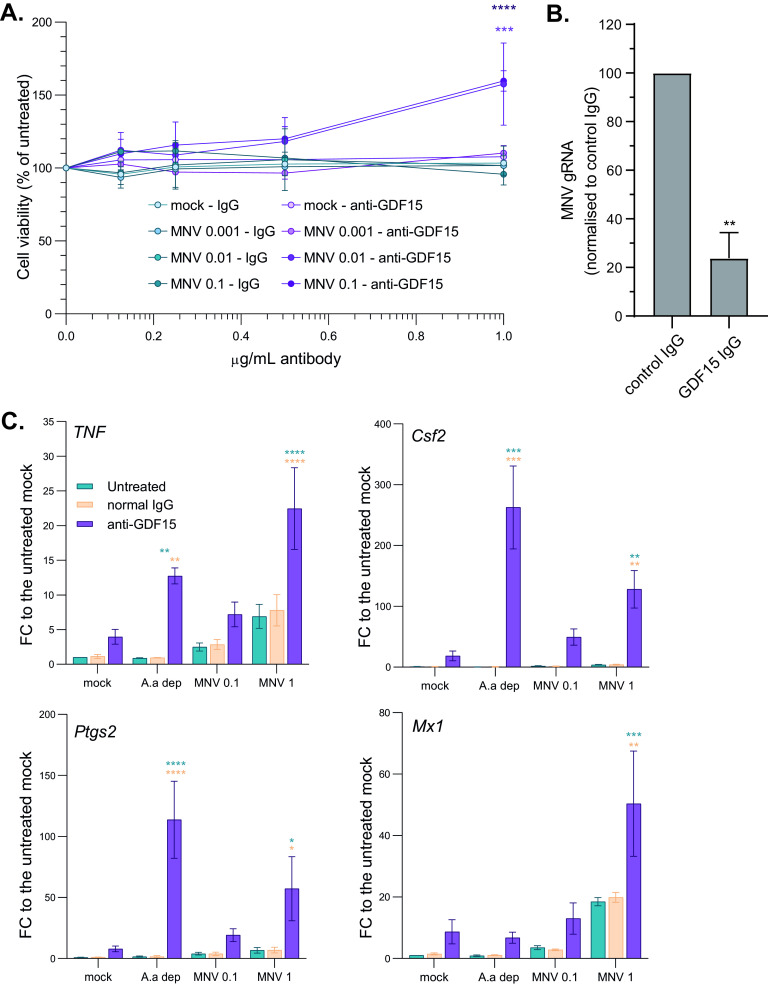
MNV infection induces the secretion of immune-tolerogenic factors. Neutralization of Gdf15 in the cell culture supernatant resulted in increased cell survival and upregulation of expression of inflammatory and antiviral factors. (A) Representative plot (*n* = 4) showing the mean results of cell viability assays performed at 30 h p.i. on RAW264.7 cells, mock-, or MNV-infected cells at an MOI of 0.01, 0.1, and 1 and incubated with normal goat IgG or anti-GDF15 antibody from 6 h p.i. onward at the indicated concentrations. Results are shown as means ± SEM, and statistical analysis results are shown above the dots. **, *P *< 0.0001; ***, *P *< 0.001. (B) MNV gRNA levels in RAW264.7 cells treated as in panel A were measured using qRT-PCR analysis. Values are means ± SEM, and statistical significance is shown above the bars (*n* = 3). ****, *P *< 0.0001; ***, *P *< 0.001; **, *P* < 0.01; *, *P *< 0.1. (C) Representative bar plots (*n* = 3) of qRT-PCR analysis of innate immune response transcripts levels. Experiment performed on total transcripts from RAW264.7 cells mock or MNV infected at an MOI of 1 for 12 h p.i. and incubated with normal goat IgG or anti-Gdf15 antibody from 6 h p.i. onward. Purified RNAs were reverse transcribed using poly(dT) primer and qPCR performed using exon-junction spanning pair of PCR primer for *Tnf*, *Csf2*, *Ptgs2*, and *Mx1* mRNAs indicated. Values are means ± SEM, and statistical significance is shown above the bars. ****, *P *< 0.0001; ***, *P *< 0.001; **, *P* < 0.01; *, *P *< 0.1.

**FIG 8:**
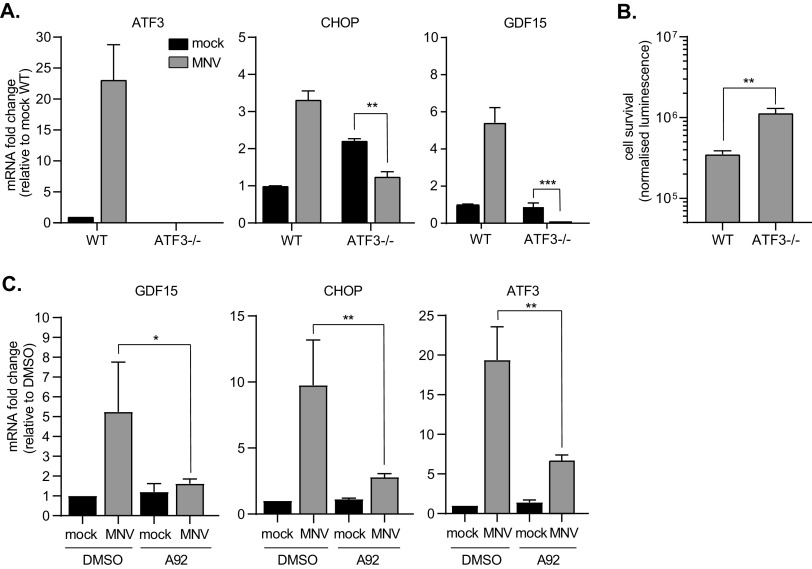
MNV-induced AAR controls GDF15 production. (A) Representative bar plots (*n* = 3) of qRT-PCR analysis of AAR signaling following ATF3 knockout. Experiment performed on total transcripts from wt or ATF3^−/s^ MEFs mock or MNV infected at an MOI of 1 for 12 h p.i. Purified RNAs were reverse transcribed using poly(dT) primer and qPCR performed using the exon-junction spanning pair of PCR primer for ATF3, CHOP, and GDF15 as indicated. Values are means ± SEM, and statistical significance is shown above the bars. ****, *P *< 0.0001; ***, *P *< 0.001; **, *P* < 0.01; *, *P *< 0.1. (B) Representative plot (*n* = 3) showing the mean results of cell viability assays performed at 12 h p.i. on wt or ATF3^−/−^ MEFs, mock, or MNV infected at an MOI of 1. Results are shown as means ± SEM, and statistical analysis results are shown above the dots. **, *P *< 0.0001. (C) Representative bar plots (*n* = 3) of qRT-PCR analysis of AAR signaling following ATF3 knockout. Experiment performed on total transcripts from mock- or MNV-infected cells at an MOI of 1 for 12 h p.i. incubated with dimethyl sulfoxide (DMSO) or the GCN2 inhibitor A-92. Purified RNAs were reverse transcribed using a poly(dT) primer, and qPCR was performed using the exon-junction spanning pair of PCR primers for ATF3, CHOP, and GDF15 as indicated. Values are means ± SEM, and statistical significance is shown above the bars. ***, *P *< 0.001; **, *P* < 0.01; *, *P *< 0.1.

Next, we addressed the propagation of anti-inflammatory signaling from MNV-infected cells to bystander cells after the first round of viral replication. In response to metabolic stress, CHOP and ATF3 upregulate the expression of GDF15, driving the M2 anti-inflammatory phenotype in macrophages (35). Given that the *Gdf15* transcript is upregulated in MNV-infected RAW264.7 cells as shown in Fig. 3B and C, we performed immunoneutralization assays to evaluate its contribution to MNV propagation. RAW264.7 cells were infected with MNV at different MOIs for 24 h, equivalent to 2 rounds of infection (12 h p.i.) and treated with 0.125 μg/ml to 1 μg/ml of GDF15-neutralizing antibody before assessing cell survival. The results showed a significant increase in cell survival at the higher dose of the neutralizing antibody compared to that of the control IgG, suggesting a correlation between MNV propagation and GDF15 activity (Fig. 7A). In addition, quantification of MNV genomic RNA (gRNA) by qRT-PCR confirmed that GDF15 neutralization resulted in 74% inhibition of viral replication (Fig. 7B). To further characterize the impact of GDF15 neutralization, cytokine levels were measured by qRT-PCR in cell cultures inoculated at an MOI of 0.1 and 1 at 12 h p.i. and after the death of the first round of MNV-infected cells (Fig. 7C). Gdf15 immunoneutralization resulted in increased levels of the proinflammatory *TNF* and *Il-6* transcripts as well as the STAT5 target genes *Csf2* and *Ptgs2* and potentiation of the paracrine type I IFN response, such as the ISG *Mx1* transcript, previously identified as a norovirus restriction factor (54). To further understand the importance of the AAR response in GDF15 upregulation during MNV infection, we engineered wild type (wt) or mouse embryonic fibroblasts (MEFs) with a homozygous genetic deletion of ATF3 (ATF3^−/−^) to make them susceptible to MNV infection by constitutively expressing the MNV receptor CD300lf as we previously did (23). Both wt and ATF3^−/−^ MEFs were infected with MNV and the activation of CHOP and GDF15 measured by qRT-PCR (Fig. 8A). These confirmed that ATF3 knockout and impaired AAR signaling impaired the MNV-induced activation of CHOP and upregulation of GDF15 while increasing cell survival (Fig. 8B). Similarly, treatment with the GCN2 inhibitor A-92, at the concentration previously shown to revert the MNV-induced stress signaling, blunted the upregulation of ATF3, CHOP, and GDF15 in MNV-infected RAW264.7 cells (Fig. 8C). Overall, this suggests that the secretion of Gdf15, downstream of ATF3/CHOP activation, results in immunosuppression and impaired antiviral paracrine signaling to promote MNV propagation.

## DISCUSSION

Previous genome-wide analyses of the transcriptome reprogramming induced during MNV replication detected perturbations in the innate immune response (40) and differences between cellular models in terms of kinetics of the host response (41). However, both studies are based on comparative analysis of uninfected cells with uninfected controls at 0 h p.i., introducing artificial sources of variation and statistical noise. Levenson et al. (41) also concluded that upregulation of *IFNβ1* transcript is uncoupled from its protein expression in RAW264.7 cells, which, together with our results on the MNV-induced translational shutoff, suggested a regulation of the antiviral response at the translation stage (19, 22, 23). Therefore, our previous identification of the activation of the eIF2α kinase GCN2 led us to readdress the global early and late host response to MNV replication using a multi-omics approach, including matched nonreplicative MNV(UVi) as a direct parallel negative control at each time point. The murine macrophage line RAW264.7 was infected at high MOI (10) and analyzed at 6 and 10 h p.i., equivalent to the first round of viral replication, to minimize the impact of paracrine immune signaling or apoptotic signaling after the death of the first infected cells (circa 12 h p.i.). Translational control analysis comparing changes in polysomal against total cytoplasmic fractions at 10 h p.i. showed a very high correlation of regulation in both fractions, which fits the known control of expression of the genes associated with the innate immune response. This result seemed to rule out any viral strategy of control at the translational level (12, 55, 56). It also demonstrated proper recruitment of the translational machinery by newly synthesized transcripts, thus confirming the absence of effect of P-eIF2α on translation efficiency during MNV replication (23).

More intriguingly, we could not identify an appropriate proinflammatory NF-κB response that would normally be instrumental in the secretion of proinflammatory autocrine and paracrine cytokines, potentiation of the IFN response, and induction of the antiviral response via activation of JAK and Stat signaling (8, 57–59). This absence of induction of an entire arm of MDA5 response is a crucial aspect of the cellular response to MNV infection, likely to be linked to its ability to replicate and propagate *in vitro* and *in vivo* (20). Remarkably, this seems coupled to the upregulation of stress-related genes such as *ATF4* and *ATF3* and their respective targets *CHOP*, *Chac1*, and *Gdf15*. We propose that these characteristics of efficient ISR signaling, downstream of GCN2 activation, and concomitant stimulation of the ATF2/c-jun in response to metabolic stress could be the key in antagonizing the antiviral inflammatory response (29, 30).

While the intrinsic apoptosis induced by MNV has been shown to favor viral replication by preventing inflammasome-related cell death (44), ATF3 is the main driver of the M2 polarization of macrophages, promoting resolution of inflammation, establishment of adaptive responses to stress, and survival (37, 38). ATF3 acts as a secondary modulator of TLR-induced inflammation *in vitro* and *in vivo* via direct transcriptional repression of NF-κB and its target genes (33, 60–62). ATF3 upregulation in response to stress, prior to LPS induction of TLR responses was shown to suppress the upregulation of *TNF* and *Il*-6 (63). This cross-tolerance is part of the immune system homeostasis to avoid excessive and prolonged inflammation. Finally, it is also responsible for the effects of nonsteroidal anti-inflammatory drugs via the upregulation of Gdf15 (35).

Time-course analysis of the activation of *ATF3* in MNV-infected RAW264.7 cells showed an upregulation concomitant to the MDA5 response, not secondary to it, which could therefore be responsible for the suppression of NF-κB response downstream of MDA5 activation. Furthermore, we showed by immunoneutralization that Gdf15, direct target gene of both ATF3 and CHOP, is responsible for a paracrine anti-inflammatory signaling in MNV-infected cell cultures. Importantly, we also demonstrate that ATF3 and induction of the AAR is essential for this MNV-induced upregulation of Gdf15. It thus appears that the activation of such a cellular response, stress homeostasis, and apoptosis may have positive effect on replication and viral fitness when triggered at the right time, before the recognition of the viral products by the cell. If it is too early, the apoptosis will prevent replication and propagation, and if it is too late, and the paracrine inflammatory signaling will block it.

The previously identified GCN2 activation along with *Asn* and *Slc1a4* gene upregulation points toward a response to amino acid starvation and metabolic imbalance in MNV-infected cells. Our characterization of the level of intracellular free amino acids by liquid chromatography-mass spectrometry (LC-MS) showed evidence of glutamine depletion as seen by its increased level compared to the mock. Glutamine is one of the critical amino acids involved in central carbon metabolism, forms a large proportion of proteins, and participates in immune cell function. Aspartate and glutamate are produced from the tricarboxylic acid (TCA) cycle metabolism and are the metabolic precursors for the biosynthesis of other proteinogenic amino acids. Increased pool sizes of these amino acids, therefore, suggested an increased metabolic activity through the TCA cycle, probably required to sustain the cellular metabolic stress and matching the MNV-induced increase of glycolysis previously described (64). It is a possibility that the unchallenged production of viral proteins, depleting the cells in both amino acids and available energy is the main factor responsible for this event. It is also noteworthy to mention that the protein VP2 contains stretches of polyglutamine that are conserved among different strains of MNV, where glutamine on its own represents 12% of amino acid usage against 3.7% in average usage in vertebrates (see Fig. S8A and B in the supplemental material). Interestingly, these polyglutamine motifs also exist in other members of the *Caliciviridae* family (Fig. S8C). It is, therefore, conceivable that the high usage of those amino acids creates elongation disruption leading to ribosome collisions and responsible for both the partial translational shutoff and activation of GCN2 in MNV-infected cells (28).

Overall, we describe the occurrence of a metabolic stress resulting from MNV replication, potentially responsible for GCN2 and ATF2 pathways activation, and the homeostatic response of the cells as driver of the host tolerance to MNV replication and propagation. The hijack of these cellular mechanisms of cross-tolerance and intrinsic apoptosis by MNV by early induction of a metabolic stress seems to be a passive event. But it may also be linked to macrophages in particular and explain the preferential tropism of MNV for cells of the myeloid lineage in addition to the presence of the CD300 MNV receptor. Finally, this strongly prompts reevaluation of the role of metabolic stress during viral infection and its impact on the cellular antiviral response. It may also offer an exciting means of increasing the infection and replication of human norovirus *in vitro* by infecting cells subsequent to a transient metabolic stress to induce a cellular tolerance to infection by incubation in amino acid-restricted medium.

## MATERIALS AND METHODS

### Cell lines and viruses.

Cells and viruses used in this study were previously described (23). Briefly, murine macrophage cells RAW264.7 and murine microglial cells BV2 were maintained in Dulbecco’s modified Eagle’s medium (DMEM), 4.5 g/liter d-glucose, Na pyruvate, l-glutamine supplemented with 10% fetal calf serum (FCS), 100 U of penicillin/ml, 100 μg of streptomycin/ml, and 10 mM HEPES (all supplements purchased from Invitrogen) at 37°C in a 5% CO_2_ environment. Murine norovirus 1 (MNV-1) strain CW1 was described previously (65) and was propagated in BV2 cells as described in reference 66 with an extra step of concentration using Amicon centrifugal filters 100k (Millipore). UV inactivation was carried by irradiation of virus-containing supernatant in a 15-cm plate prior to the concentration step in Stratalinker 1800 (Stratagene) at the standard power for 3 times 3 min with swirling of the plate between each round. Virus titers were estimated by determination of the TCID_50_ in units per milliliter in RAW264.7 cells, and infections were carried at an MOI of 10 unless stated otherwise. The times postinfection refer to the time elapsed following medium replacement after a 1-h inoculation period at 37°C in a 5% CO_2_ environment. MEFs wt and ATF3^−/−^ expressing the MNV receptor CD300lf were produced by retroviral transduction of MEF wt and ATF3^−/−^ (kind gift of T. Hai, Ohio State University) with the lentiviral particles containing the full-length CD300lf sequence and hygromycin resistance gene, selected at 100 μg/ml of hygromycin B (Sigma) and propagated in complete medium containing 50 μg/ml of hygromycin B as described before (23). TLR3 antiviral response was elicited by addition of the synthetic double-strand RNA poly(I·C) (reconstituted at 10 mg/ml in phosphate-buffered saline (PBS) by incubation at 50°C; Sigma) at 20 μg/ml directly into the cell medium for the indicated times. For the starvation experiment, cells were washed twice in glutamine-, cysteine-, and methionine-depleted medium (Invitrogen; hereafter called GCM-dep medium) and 4.5 g/liter d-glucose, supplemented with 10% FBS, 10 mM HEPES, 1 mM Na Pyruvate before incubation for the indicated times in the same medium. GCN2 inhibition was performed by adding A-92 (Axon Medchem) at 500 nM to the cell medium for the indicated times. JNK2 inhibition was performed by adding SP6000125 (Tocris) at 20 μM to the cell medium for the indicated times.

### Polysome fractionation and RNA sequencing.

Polysome fractionation has been performed as described previously (21). Briefly, 10^7^ RAW264.7 cells uninfected or infected with MNV(UVi) or MNV at an MOI of 10 for 6 and 10 h were treated with 100 μg/ml of cycloheximide (CHX) (Sigma) for 3 min at 37°C to lock the translating ribosomes onto the transcripts before being placed on ice, washed twice with cold PBS (Invitrogen) containing 100 μg/ml CHX and lysed on plate with 1 ml of cold lysis buffer (15 mM Tris-HCl, pH 7.5, 300 mM NaCl, 15 mM MgCl_2_, 100 μg/ml CHX, 1% [vol/vol] Triton X-100, and 200 U RNasin [Promega] in RNase-free water [Qiagen]). Lysis was carried on for 5 min, and the lysates were transferred to microcentrifuge tubes and spun at 10 krpm for 3 min at 4°C to remove nuclei. The supernatants were aliquoted in 2 tubes for total and polysomal RNA isolation. Five hundred microliters of each lysate was loaded onto 10 ml 10 to 60% discontinuous sucrose gradients (15 mM Tris, pH 7.5, 300 mM NaCl, 15 mM MgCl_2_, 100 μg/ml CHX, 1 mg/ml heparin) and spun at 38 krpm for 2 h at 4°C using the SW 41 Ti rotor (Beckmann). Gradients were fractionated using the fraction collector Foxy R1 (Teledyne ISCO, Lincoln, NE) in 10 fractions of 1 ml in 15-ml tubes, and UV absorbance was monitored at 254 nm, recorded using the PeakTrail software and plotted using GraphPad. Total lysates and gradient fractions were denatured in 1.5 ml of 7 M GnHCl, mixed vigorously and precipitated with 2 ml of 100% ethyl alcohol (EtOH) at −80°C overnight. RNAs were pelleted at 4,000 rpm for 50 min at 4°C before being resuspended in 400 μl of RNase-free water, transferred to microcentrifuge tubes, and reprecipitated with 40 μl of 3 M NaOAc, pH 5.2, and 1 ml of 100% EtOH at −80°C overnight. Samples were centrifuged at top speed in a microfuge for 30 min at 4°C, washed once with 75% EtOH, air dried, and resuspended in 30 μl of RNase-free water before quantification. The fractions containing the polyribosomes were identified by their composition in 40 and 60S rRNA on 2% agarose gel, pulled together, and purified with LiCl precipitation by addition of an equal volume of 7.5 M LiCl solution (Ambion), incubation overnight at −20°C, spun at top speed for 30 min at 4°C, washed in 75% EtOH, resuspended in 50 μl before a second round of LiCl precipitation, and eventually resuspended into 30 μl of RNase-free water. Three independent replicates of the experiment were performed before control of the purified RNA quality on Bioanalyzer (Agilent) as per the manufacturer’s instructions and quantification on Nanodrop. One microgram of RNA per sample was sent to the Oxford Genomics Centre (Welcome Trust Centre for Human Genetics, Oxford, UK), which generated the cDNA libraries with enrichment in poly(A)-tailed mRNA and performed the RNA sequencing on an Illumina HiSeq 4000 machine with a 75 bp PairEnds protocol with multiplexing of the samples.

### Analysis of RNA sequencing data.

RNA sequencing reads were quality checked via FastQC (v 0.11.4). Reads were mapped to the mouse genome (GRCm38 reference genome, Hisat2 prebuilt index files downloaded from ftp://ftp.ccb.jhu.edu/pub/infphilo/hisat2/data/grcm38_tran.tar.gz) using Hisat2 (v 2.0.5) (67) and to the murine norovirus genome MNV1-CW1 (GenBank accession number DQ285629) coding sequences (ORF1, GenPept accession number ABB90153.1; ORF2, GenPept accession number ABB90154.1; ORF3, GenPept accession number ABB90155.1) using Bowtie 2 (v 2.2.5) (68). The function ‘featureCounts’ (69) from the R Bioconductor package ‘Rsubread’ (v 1.16.1) was used to assign mapped sequencing reads to genome features. Genomic features of the host were defined by the tool’s built-in gene annotations for the mouse genome (NCBI RefSeq gene annotations build 38.1). Host genomic features were annotated using the R package ‘org.Mm.eg.db’ (v 3.7.0), Ensembl (accessed via the R package ‘biomaRt’ [v 2.38.0]), and GenBank (accessed via the R package ‘Annotate’ (v 1.52.1). The feature count matrix consisted of the set of host and MNV feature count, and genes annotated as rRNA, microRNA (miRNA), snoRNA, or small Cajal body-associated RNA (scaRNA) were filtered out of the feature count matrix. Filtering of lowly expressed genes was performed after library size normalization by keeping genes with at least 0.25 count per million (CPM) in at least 25% of the samples in both total and polysomal fractions. Correlation and PCA analysis had been made on the resultant log_2_(CPM) matrix. The degree of correlation between replicates on the Hisat2/Bowtie was analyzed on respective complete feature count matrix using the R function ‘cor’ with the Pearson method and complete linkage hierarchical clustering using the function embedded within the plot generating R CRAN package ‘superheat’ (70). PCA analyses were completed using either the R function ‘pca’ (71) or the R CRAN package ‘pca3d’ (72). Differential expression analysis was performed using the EdgeR quasi-likelihood (QL) pipeline (73, 74). Briefly, after scaling normalization by library size and compositional normalization using the trimmed mean of M-values (TMM), count data were fitted to a quasi-likelihood negative binomial-generalized log-linear model. Differential expression in selected contrasts was identified using gene-wise empirical Bayes quasi-likehood F-tests. The Benjamini-Hochberg (BH) procedure was used for multiplicity correction of *P* values, and a threshold of significance at a BH *P* value of <0.1 was selected (75). A final filtering criterion was applied to each individually significant gene using the open-source genome visualization tool Integrative Genome Viewer IGV hosted by the Broad Institute (https://software.broadinstitute.org/software/igv/home) (76), and genes displaying poor or aberrant coverage were discarded. Heatmaps of the significant differentially expressed genes were generated on centered and scaled log_2_(CPM) for each gene using the ‘cor’ function with the Pearson method and represented with the ‘superheat’ R package after hierarchical clustering. Translational control analysis was completed using the Bioconductor package Anota2seq (39) with default parameters. Genome ontology (GO) enrichment was performed on the web-based software Metascape (https://metascape.org/gp/index.html#/main/step1) (77) and on the open-source software platform Cytoscape (v 3.8.0) (42) using the plug-in ClueGO (78) with the following query settings: GO_Biological Process and KEGG_pathway with pval < 10^−5^ only and a *fair* Kappa score of connectivity. The statistical significance of the enrichment was measured using the hypergeometric test implemented with the Bonferroni stepdown correction of the *P* values and grouping of the GO term based on the highest significance (Kappa score). The corresponding annotations network was generated using a prefuse force directed layout setting. All other charts were generated on GraphPad (v 8.2.1). Transcription network enrichment was analyzed using the WEB-based Gene SeT analysis toolkit (http://www.webgestalt.org/) (79) using the overrepresentation analysis method and the transcription factor target database MSigDB (v 6.2) (80). Comparison between studies or cellular backgrounds results were made on the web-based integrative tool for comparing lists JVenn (http://jvenn.toulouse.inra.fr/app/index.html) (81).

### Phosphoantibody array.

Assays were conducted as described in reference 21. Briefly, 300 μg of protein from mock- and MNV-infected RAW264.7 cells lysed at 2 and 12 h p.i. were analyzed using the Proteome Profiler Human Phospho-MAPK array (R&D Systems). The signal was detected on radiographic film (Fuji RX) and quantified using Image J software.

### RNA purification and qRT-PCR.

Approximately 2.10^6^ RAW264.7 cells were plated onto 35-mm dishes and infected the next day with MNV at an MOI of 10 as described above. Cells were washed once with cold PBS, lysed on plate using 500 μl of lysis buffer from the Quick-RNA miniprep kit (Zymo Research), and RNA subsequently isolated using the same kit according to the manufacturer’s instructions before quantification on Nanodrop. Reverse transcriptions were carried out on 0.5 to 1 μg of purified RNA using the Precision nanoScript2 reverse transcription kit (Primer Design), and qPCR was performed on 5 μl of the cDNA libraries diluted at 1:10 using the Precision Plus 2× qPCR mastermix (Primer Design) according to the manufacturer’s instructions and the Quant Studio 7 Flex (Applied Biosystems). For validation of the translatomic results, RT-qPCR was carried out on the RNA sequencing samples as mentioned above, and results were plotted against each other in Graph Pad. Correlation coefficient was calculated on grouped polysomal and total data using the Pearson method in GraphPad. Primers are listed in the supplementary data.

### Biorthogonal labeling for characterization of *de novo* proteome.

Stable isotope labeling of amino acids in RAW264.7 cell culture was performed as described in reference 82 and carried out in high-glucose DMEM lacking arginine and lysine (Sigma-Aldrich) supplemented with dialyzes 10% FBS, 1% l-glutamine, 1× non-essential amino acid (NEAA), 10 mM HEPES, and 1× penicillin/streptomycin. RAW264.7 cells were maintained in SILAC medium supplemented with light (R0K0), medium (R6K4), or heavy (R10K8) arginine and lysine (Cambridge Isotope Laboratories) for 5 passages, ensuring complete incorporation of the different isotopes. For labeling of the neotranslatome, 10^7^ SILAC-labeled cells were either mock, MNV(UVi), or MNV infected at an MOI of 10 TCID_50_/cell before replacing with SILAC medium without methionine for 30 min at 37°C at 5.5 h and 9.5 h p.i., and then labeled with SILAC medium containing 1 mM l-azidohomoalanine (AHA; Dundee Cell Products) (53, 83) and incubated for 30 min at 37°C prior to lysis with 2% SDS in PBS-containing 5 U/μl benzonase (Sigma). Cell lysates were heated for 10 min at 95°C and centrifuged at 21,000 × *g* for 10 min at 4°C. The supernatant was collected as total cell lysates, and 10% of each sample was kept aside as total cell input. After concentration normalization using bicinchoninic acid (BCA) analysis (Pierce) following manufacturer instructions, 1.5 mg total cell lysates were diluted to 0.2% SDS, 0.2% Triton X-100 in PBS, pH 7.4/PI-E, depleted of endogenous biotinylated proteins by incubation with 30 μl streptavidin beads (Pierce 88817) overnight at 4°C before click reaction with 100 μM biotin-alkyne, 500 μM THPTA (Dundee Cell Products), 100 μM CuSO_4_, 5 mM aminoguanidine, and 2.5 mM sodium ascorbate, and the reaction was allowed to proceed for 6 h at room temperature before removal of the biotin excess using Zeba desalting columns (7 kDa cutoff; Thermo Fisher). The samples were then combined altogether at a 1:1:1 ratio within one replicate. Three independent replicates were made with a different SILAC medium assigned to a given condition for each replicate. AHA-labeled biotinylated peptides were affinity enriched by incubation with 300 μl streptavidin beads (Pierce) overnight at 4°C with rotation in PBS containing 0.1% SDS (vol/vol) and washed 20 times with 0.5 ml cold PBS 1% SDS with complete removal of the liquid before elution of the bound fractions in 75 μl PBS 2% SDS, 1 mM Biotin, and denaturation at 95°C for 5 min. Validation IP was performed similarly, but the AHA-labeling time was increased to 2 h to improve labeling and enrichment. Eluted fractions were submitted for mass spectrometry analysis at the University of Bristol Proteomics Facility as described in reference 82. SILAC pooled samples were run on an SDS-PAGE gel, and each gel lane was cut into 10 equal slices. Each slice was subjected to in-gel tryptic digestion using a DigestPro automated digestion unit (Intavis Ltd.) to minimize manual handling. The resulting peptides were fractionated using an Ultimate 3000 nano-LC system in line with an Orbitrap Fusion Tribrid mass spectrometer (Thermo Scientific). In brief, peptides in 1% (vol/vol) formic acid were injected onto an Acclaim PepMap C_18_ nano-trap column (Thermo Scientific). After washing with 0.5% (vol/vol) acetonitrile, 0.1% (vol/vol) formic acid peptides were resolved on a 250 mm by 75 μm Acclaim PepMap C_18_ reverse-phase analytical column (Thermo Scientific) over a 150-min organic gradient, using 7 gradient segments (1 to 6% solvent B over 1 min, 6 to 15% B over 58 min, 15 to 32% B over 58 min, 32 to 40% B over 5 min, 40 to 90% B over 1 min, held at 90% B for 6 min, and then reduced to 1% B over 1 min) with a flow rate of 300 nl min^−1^. Solvent A was 0.1% formic acid, and solvent B was aqueous 80% acetonitrile in 0.1% formic acid. Peptides were ionized by nano-electrospray ionization at 2.0 kV using a stainless-steel emitter with an internal diameter of 30 μm (Thermo Scientific) and a capillary temperature of 275°C.

All spectra were acquired using an Orbitrap Fusion Tribrid mass spectrometer controlled by Xcalibur 2.1 software (Thermo Scientific) and operated in data-dependent acquisition mode. FTMS1 spectra were collected at a resolution of 120,000 over a scan range (*m/z*) of 350 to 1,550, with an automatic gain control (AGC) target of 3E5 and a max injection time of 100 ms. Precursors were filtered using an intensity range of 1E4 to 1E20 and according to charge state (to include charge states 2 to 6) and with monoisotopic precursor selection. Previously interrogated precursors were excluded using a dynamic window (40 s ± 10 ppm). The MS2 precursors were isolated with a quadrupole mass filter set to a width of 1.4 m/z. ITMS2 spectra were collected with an AGC target of 2E4, max injection time of 40 ms, and collision-induced dissociation (CID) energy of 35%.

### Analysis of SILAC-AHA proteomics data.

The raw data files were processed and quantified using MaxQuant software v 1.6.1.0 and searched against the UniProt mouse database (downloaded 30 March 2018, 61,314 entries) and MNV1 protein sequences (GenBank accession number DQ285629.1). Peptide precursor mass tolerance was set at 20 ppm, and tandem mass spectrometry (MS/MS) tolerance was set at 0.5 Da. Search criteria included oxidation of methionine (+15.9949), acetylation of protein N termini (+42.0106), Triazole-PEG4-biotin of methionine (+452.238 Da), AHA of methionine (−4.9863), and SILAC labels (+6.020 Da and +10.008 Da at arginine and +4.025 Da and +8.014 Da at lysine) as variable modifications. Searches were performed with full tryptic digestion, and a maximum of 2 missed cleavage was allowed. The reverse database search option was enabled, and the data was filtered to satisfy a false discovery rate (FDR) of 1%.

Differential expression was analyzed by computing the pairwise ratios of either pair of mock-infected or UVi(MNV) and MNV-infected samples, and the Log_2_SILAC ratios for proteins identified in at least 2 out of 3 replicates were averaged and proteins considered significantly regulated if the pairwise ratio was in the top or bottom 2.5% either contrasting MNV/mock or MNV/UVi(MNV). Subsequent computational analysis had been made as described in “Analysis of RNA sequencing data.” The results were validated against the total inputs by immunoblotting for some of the major proteins identified as significantly enriched in the *de novo* proteome at 6 and 10 h p.i.

### Immunoblotting.

Immunoblotting analysis was performed as described in reference 23. Briefly, approximately 2.10^6^ RAW264.7 cells were plated onto 35-mm dishes and infected the next day with MNV at an MOI of 10 as described above. At the indicated times, cells were lysed in 150 μl of 1× gel loading buffer (New England BioLabs), sonicated, and boiled 5 min at 95°C. Cell lysates were separated by SDS-PAGE using 10 μg of total proteins, and the proteins were transferred to polyvinylidene difluoride membranes. These were then probed with the following primary antibodies: mouse anti-phospho-ATF2 T71 (1:1,000; Abcam), rabbit anti-ATF3 (1:500; Cell Signaling), goat anti-TNF-α (1:500; Santa Cruz), rabbit anti-Ddx58 (1:500; Santa Cruz; sc-376845), rabbit anti-Stat1 (1:1,000; Abcam; Ab92506), rabbit anti-ATF2 (1:1,000; Cell Signaling; 35031), mouse anti-VP1 (1:500; I. G. Goodfellow, Cambridge, UK), anti-MNV-3 mouse immune sera (1:1,000; I. G. Goodfellow, Cambridge, UK), rabbit anti-NS7 (1:10,000; I. G. Goodfellow, Cambridge, UK), mouse anti-glyceraldehyde-3-phosphate dehydrogenase (GAPDH) (Clone 6C5, 1:20,000; Invitrogen), followed by incubation with the appropriate peroxidase-labeled secondary antibodies (Dako) and chemiluminescence development using the Clarity Western ECL Substrate (Bio-Rad). The results were acquired using the Vilber imaging system.

### Quantification of total amino acids.

The 2.10^6^ RAW264.7 cells were plated on 35-mm plates and mock or MNV infected the next day at an MOI of 10 or washed once and incubated in GCM-dep medium instead of the normal medium for the cells in starvation control. At 10 h p.i., cells were placed on ice, scraped in cold PBS, transferred to a 1.5-ml tube, washed one more times in cold PBS, and the pellets kept at −80°C. The pellets were then resuspended in 300 μl of cold l-amino acid assay buffer according to the manufacturer’s instructions (l-amino acid quantification kit; Sigma), and lysates were then centrifuged at 13,000 rpm for 10 min at 4°C to remove insoluble material. The supernatants were transferred to a new tube, and the amino acids concentrations measured in duplicate on 50 μl of each lysate by adding one volume of the master reaction mix and 30 min incubation at 37°C in the dark. Results were measured at 570 nm in Clariostar plate reader against the kit control standard curve and normalized by the protein contents quantified using the BCA kit (Pierce) according to the manufacturer’s instructions.

### Quantification of free individual amino acids.

The 6.10^6^ RAW264.7 cells per condition were plated onto 15-cm plates and treated as mentioned above. At 10 h p.i., metabolites were harvested by collecting the cells in cold PBS, followed by two washes in cold PBS, resuspension in 400 μl of Triton X-100 0.1% (vol/vol) in LC-MS-grade water (Fisher), and lysis in 2:1 methanol (400 μl)/chloroform (200 μl) (LC-MS grade Fisher). Lysates were mixed well and incubated for 10 min on ice. The lysates were then extracted by incubation for 10 min at room temperature (RT) before phase separation by centrifugation at 8 krpm for 10 min at RT. Metabolites were separated into polar and nonpolar phases. The free amino acids were recovered in the polar phase and used for the metabolomics analysis by gas chromatography-mass spectrometry (GC-MS). The intermediate phases containing the proteins were used for determination of the protein content after spinning down at full speed for 10 min, complete removal of the liquid phases, air-drying for 5 min before lysis in 300 μl of 0.1 N NaOH, and sonicated until clear. Quantification of the protein contents was made using the BCA kit according to the manufacturer’s instruction. Three independent replicates were completed before being further processed by GC-MS. Authentic standards for 21 amino acids were purchased from Sigma-Aldrich and were used for method development. Norvaline (Sigma-Aldrich) was used as the internal standard for GC-MS analysis and for data normalization. Five microliters of 10 ng · μl^−1^ norvaline was added to the extracted polar phase and dried. Standards and dried metabolite extracts were mixed with 25 μl pyridine and incubated at 900 rpm, 37°C, and for 30 min. Thirty-five microliters tert-butyldimethylsilyl chloride (TBDMSCl) (Sigma-Aldrich) was added to the pyridine-metabolite extracts and mixed at 900 rpm, 60°C, and for 30 min. After this incubation, samples were briefly spun, transferred to GC-MS vials, and sealed. One microliter of the derivatized metabolites were injected into an injection port of GC 7890 system temperature maintained at 230°C. Amino acids were separated using a VF-5ms inert 5% phenyl-methyl column (Agilent Technologies). The oven temperature was constant at 120°C for 5 min, followed by an increased to 270°C at 4°C/minute and was held at this temperature for 3 min. The temperature was further increased to 310°C at 20°C/minute and was held at this temperature for 1 min. The carrier gas was helium, and the flow was maintained at 1.3 ml/minute. The total run time of each sample was 51.667 min, including a solvent delay of 10 min. The scan parameters for the MS detector were set to 150 for low mass and 600 for high mass. MS data was detected using electron ionization (EI) on a 5795 MS system with triple axis detector (Agilent Technologies). The scan parameters for the MS detector were set to 50 for a low mass and 600 for a high mass. Identification of free amino acids was done in AMDIS (Automated Mass Spectral Deconvolution and Identification System), developed at the National Institute of Standards and Technology (NIST), and free amino acids were identified by their characteristic fragmentation patterns and *m/z* values as previously published (84). GC-MS data was extracted using the ChemStation software (Agilent Technologies) and detection and quantitation parameters previously established (84). Total ion counts for the M-57 *m/z* fragments from 21 amino acids were extracted for quantitation.

### Cell viability assay.

RAW264.7 or MEF cells were mock or MNV infected at an MOI of 0.1, 0.01 or 0.001 in complete medium, and 5.10^3^ cells in 100 μl/well were plated onto a white-walled and clear-bottom 96-well plate (Costar) in duplicate. Neutralizing goat antibody against human GDF15 or normal goat IgG (R&D systems) was added at 6 h p.i. at 0.125, 0.25, 0.5, and 1 μg/ml and the cells incubated until 24 h p.i., ensuring 2 rounds of infection, and the plates were kept at −80°C until further processing. Cell viability was assessed using the CellTiter-Glo 2.0 assay (Promega). Briefly, 100 μl of room temperature reagent was added to each well on ice and mixed by pipetting. The plates were then incubated at room temperature for 1 h before reading of the luminescence on the BMG microplate reader (Labtech) using the Clariostar software with an integration time of 2 s. The results were blank adjusted and normalized against the untreated cells of the same condition.

### Data availability.

The raw RNA-Seq data and mapping results can be found as part of GEO repository database under accession number GSE168402. The raw proteomic data, search results, and FASTA file can be found in the PRIDE repository database under project number PXD025169 (https://www.ebi.ac.uk/pride/archive/projects/PXD025169).
