# Supplementary material for: Murine Norovirus Infection Results in Anti-inflammatory Response Downstream of Amino Acid Depletion in Macrophages
Source: J Virol. 2021 Sep 27;95(20):e01134-21. doi: 10.1128/JVI.01134-21 (PMC8475529; doi:10.1128/JVI.01134-21)
Supplement: Supplemental file 1 — Fig. S1 to S8. Download JVI.01134-21-s0001.pdf, PDF file, 5.2 MB [file jvi.01134-21-s0001.pdf]

Supplementary Figure 1

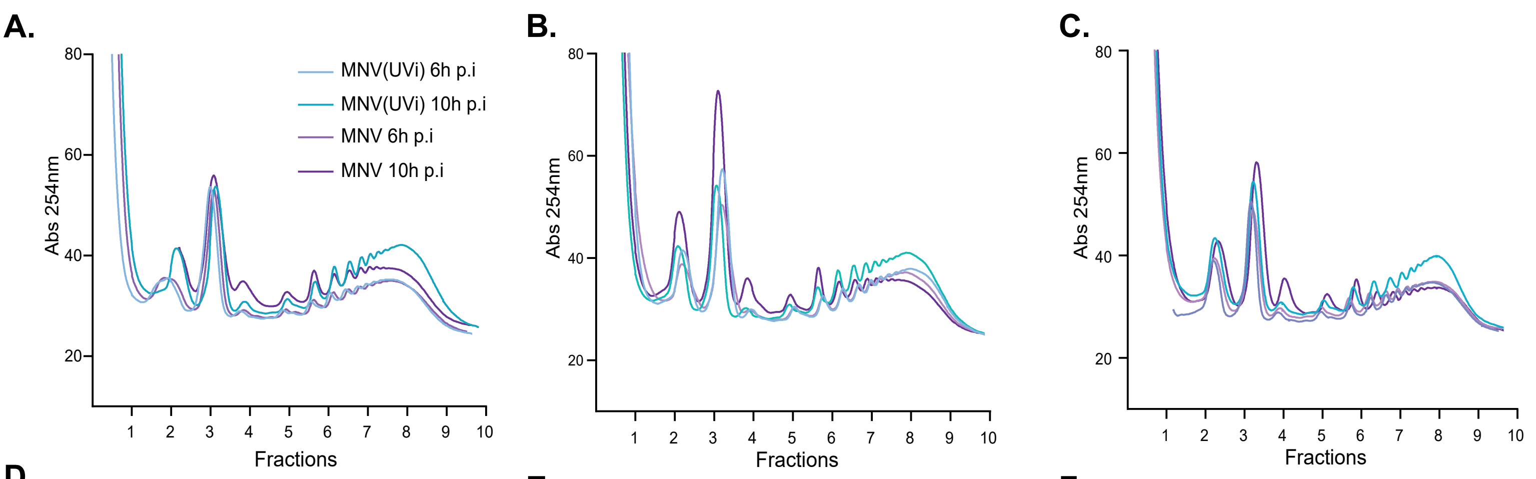

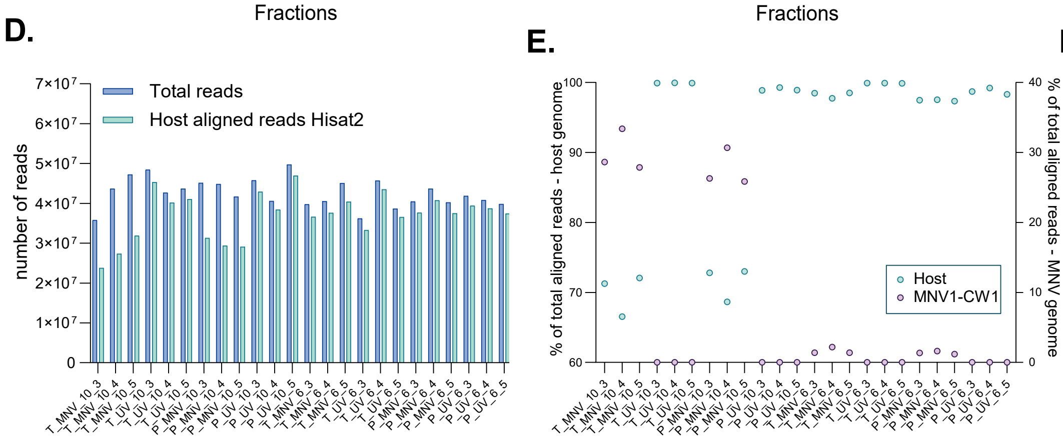

F.

	Hisat2 complete	Hisat2 filtered
all gene	17912	17736
protein coding	15237	15237
pseudogenes	508	508
ncRNA	1690	1642
snRNA	15	0
snoRNA	110	0
rRNA	3	0
other	17	17
unknown	332	332

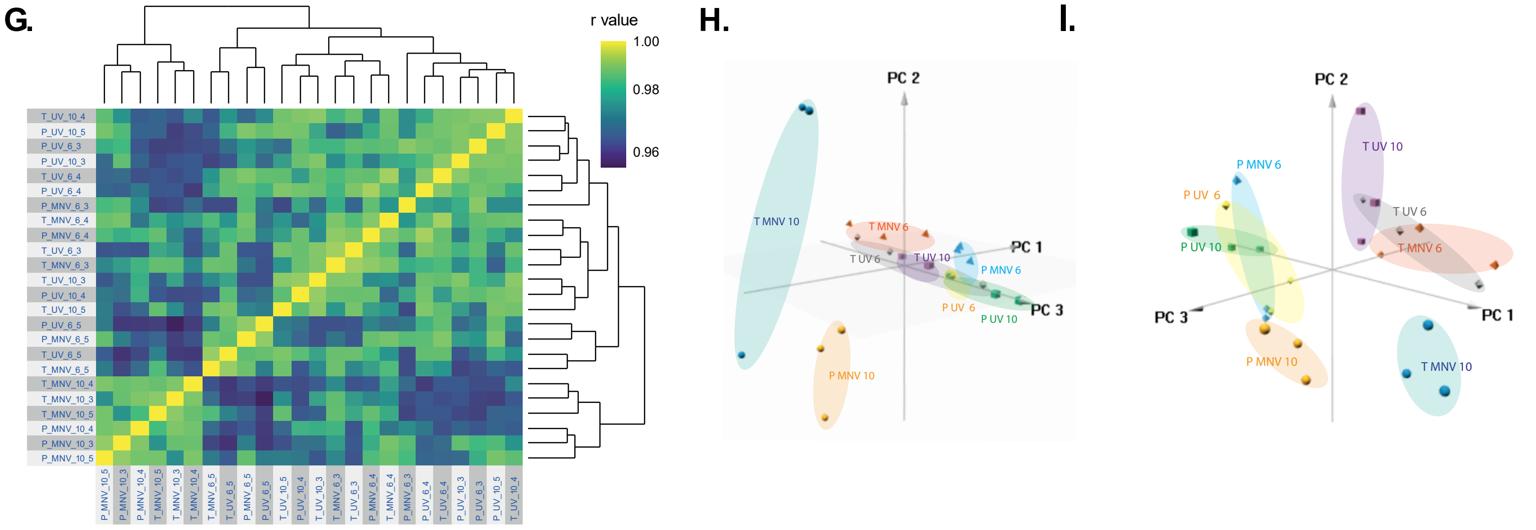

S1 Fig: Polysomes fractionation (A-C) and exploratory analysis of the RNA sequencing data in RAW264.7 cells (D to I). (A-C) Absorbance profiles of the total cytoplasmic extracts fractioned onto 10 to 50% sucrose gradients, monitored at 254nm and recorded on PeakTrail for the three biological replicates used in this work. MNV(UVi) 6h p.i. (light blue), MNV(UVi) 10h p.i. (cyan), MNV 6h p.i. (light purple), MNV 10h p.i. (deep purple). Fractions 1 to 2, free material; fractions 2 to 4, free ribosomal subunits 40 and 60S and monosomes 80S; fractions 5 to 10, polysomal fractions. Note the decrease in polysomal material in MNV 10h p.i. samples compared to the MNV(UVi) counterpart matching the translation shutoff previously observed in MNV-infected cells after 6h p.i. (22). (D) Bar plot of the total number of reads for each sample (blue bars) and corresponding number of reads mapped to the host genome (GRCm38.p5 murine genome primary assembly) using Hisat2 (green bars) software. (E) Scatter plot showing the percentage of total reads mapped to the murine genome using either Hisat2 (blue dots, left y axis) for each sample and the subsequent percentage of reads mapping to MNV1-CW1 genome (DQ285629) (purple dots, right y axis). (F) Mapped reads using Hisat2 were assigned a genomic feature (complete dataset) subsequently annotated using GenBank. All genes annotated as rRNA, miRNA, snoRNA, scaRNA were filtered out (filtered dataset). (G) Analysis of the batch effect of the biological replicates on the RNA sequencing outputs measured by calculation of the pair correlation of all samples from the complete Hisat2 (Host) and Bowtie (MNV) dataset, represented as a matrix. Correlation calculation using the Pearson method on log2CPM of the complete dataset filtered for low reads genes and small non-coding RNA, coupled with complete linkage hierarchical clustering showing the low impact of the batch effect with high correlation for samples of the same condition across the three replicates (average r = 0.979 ± 0.007) and highlighting the multiple sources of variation between the samples as infection, time post-infection and total or polysomal fraction. (H – I) 3D representation of principal component analysis of the complete Hisat2 (host) and Bowtie (MNV) dataset (H) or the Hisat2 only (host) dataset (I) showing the combinatorial order of the variation parameters in presence or absence of the viral RNA in the dataset. Principal components scores were calculated on log2CPM datasets filtered for low reads genes and small non-coding RNA. 3D cluster plot of the results showed grouping of the samples following the variables identified by correlation analysis and a clear separation of the samples from MNV-infected cells lysed at 10h p.i. independently of the occurrence of the viral RNA in the dataset, highlighting the combination “infection” and “10h p.i.” as the main sources of variation in this experiment.

Supplementary Figure 2

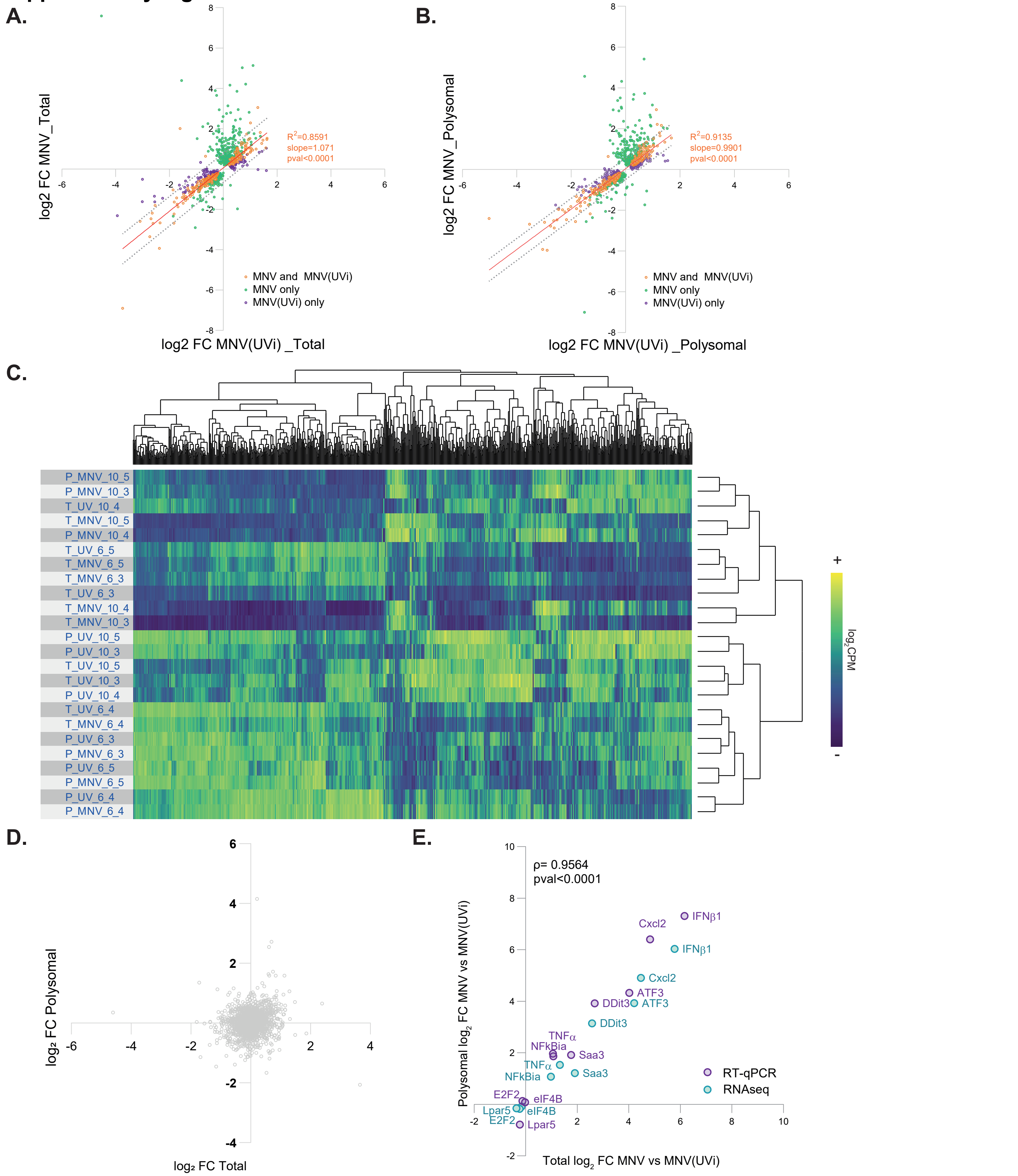

S2 Fig.: Differential expression analysis of MNV-infected cells compared to MNV(UVi)-infected cells in overtime comparison exposed wide array of changes common to both conditions. Scatter plot of the results of the multivariate analysis of the differential expression comparing the 10 vs 6h p.i. time points for the MNV-infected cells vs the MNV(UVi)-infected cells in the Total fraction (A) and the Polysomal fraction (B). Results were expressed as log2 (FC 10vs6) and gene changes were considered significant when associated with a BHpval <0.1. MNV and MNV(UVi) results were merged, omitting genes identified in only one condition, and their respective log2(FC) for the significant dataset were used in the biorthogonal projection, MNV-infected cells (y axis) vs MNV(UVi)-infected cells (x axis). Genes were further subdivided in 3 groups: significant in MNV-infected cells only (green dot), significant in MNV(UVi)-infected cells only (purple dot), significant in both (orange dot). (A) Comparison in the total fraction showing positively correlating regulated genes in both contrasts (linear regression, R2=0.9135, pval<0.0001). The apparent trend of genes regulated only in MNV(UVi) clustering in and near the 95% confidence prediction bands of linear regression from the genes regulated in both contrasts suggested a stringent significance threshold rather than an actual difference between the conditions. The genes regulated in MNV-infected cells only can be subdivided into 3 clusters: in and around the IC95%, an important cluster of upregulated genes and a small cluster of downregulated genes, both likely to be MNV-specific. (B) Same comparison than in A. for the RNA from the Polysomal fraction. (C) Heatmap of the comparison of the significant differentially expressed genes in MNV-infected cells overtime (green dots in A. and B.) displaying their log2CPM values in each sample showing an absence of strict clustering following infection, time, fraction or replicate suggesting the predominance of uncontrolled variability sources and unsuitability of this analysis design. Values were centred and scaled before hierarchical clustering using Pearson method and complete linkage hierarchical clustering. (D) Scatter plot of the differential expression analysis of the contrasts MNV-infected cells vs MNV(UVi)-infected cells at 6h p.i. in Total (x axis) vs Polysomal (y axis) fraction showing no significant changes. (E) Validation by qRT-PCR of the translatomic results at 10h p.i. Scatter plot of the log2FC from the differential expression analysis (green dots) for the contrasts MNV-infected cells vs MNV(UVi)-infected cells at 10h p.i. in Total (x axis) vs Polysomal (y axis) fraction compared with the log2FC obtained by qRT-PCR (purple dots) showing a high degree of correlation (Spearman ρ= 0.9564, pval<0.0001).

Supplementary Figure 3

A.

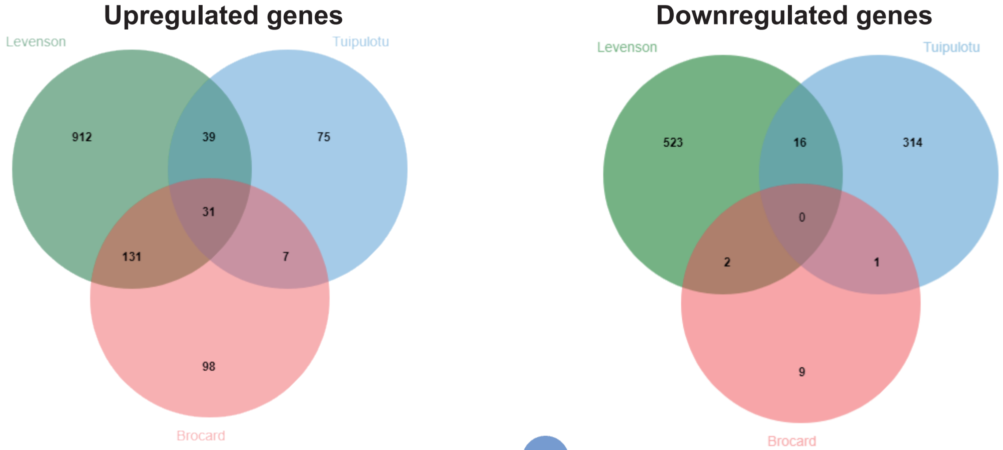

B.

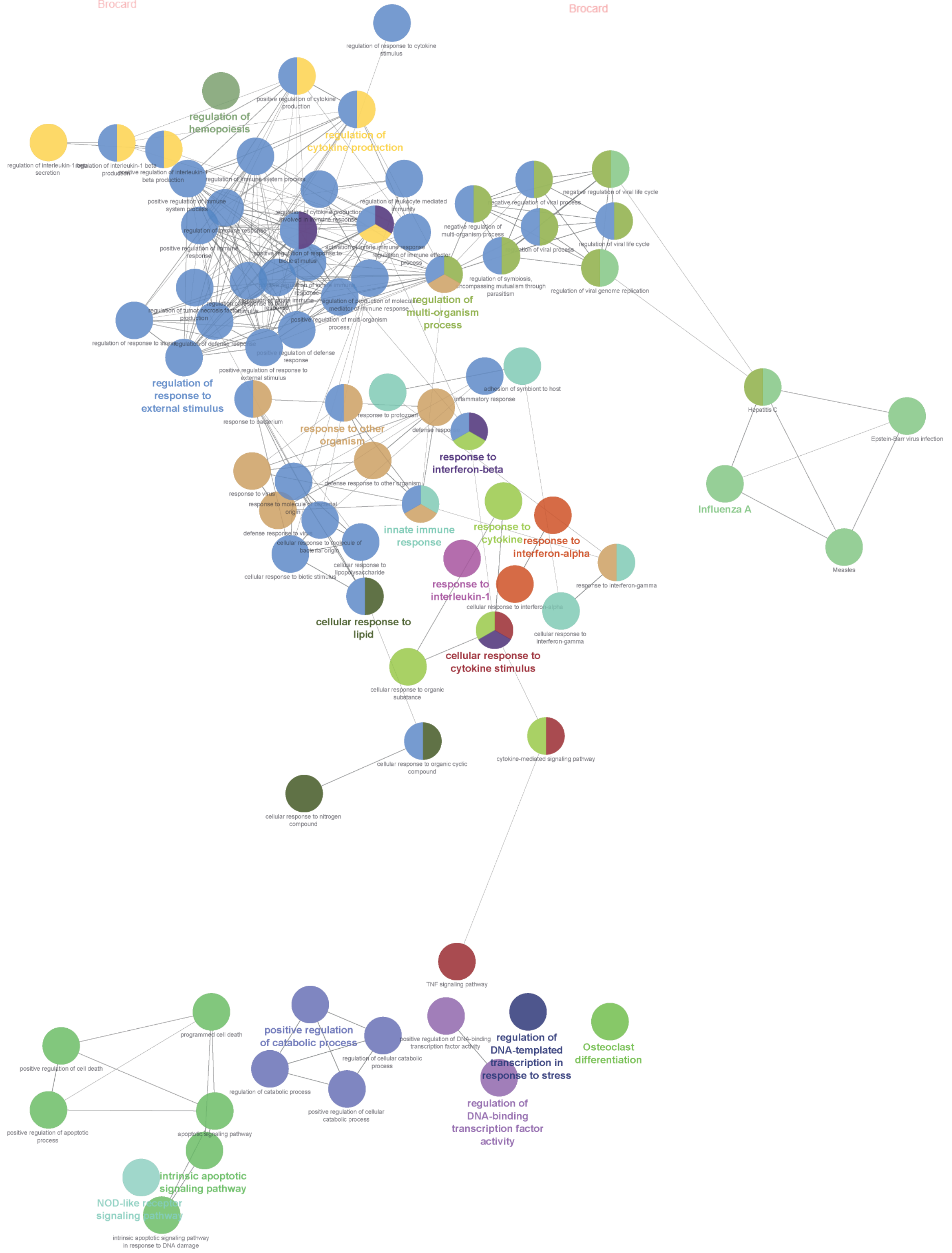

S3 Fig.: MNV-induced upregulated genes belong to the IFN response and to the cellular response to stress linked to intrinsic apoptotic pathway. Annotation network of the GO analysis of the genes significantly regulated in MNV-infected cells (Total and Polysomal) at 10h p.i. as presented in Fig. 2F and generated using a prefuse force directed layout setting.

Supplementary Figure 4

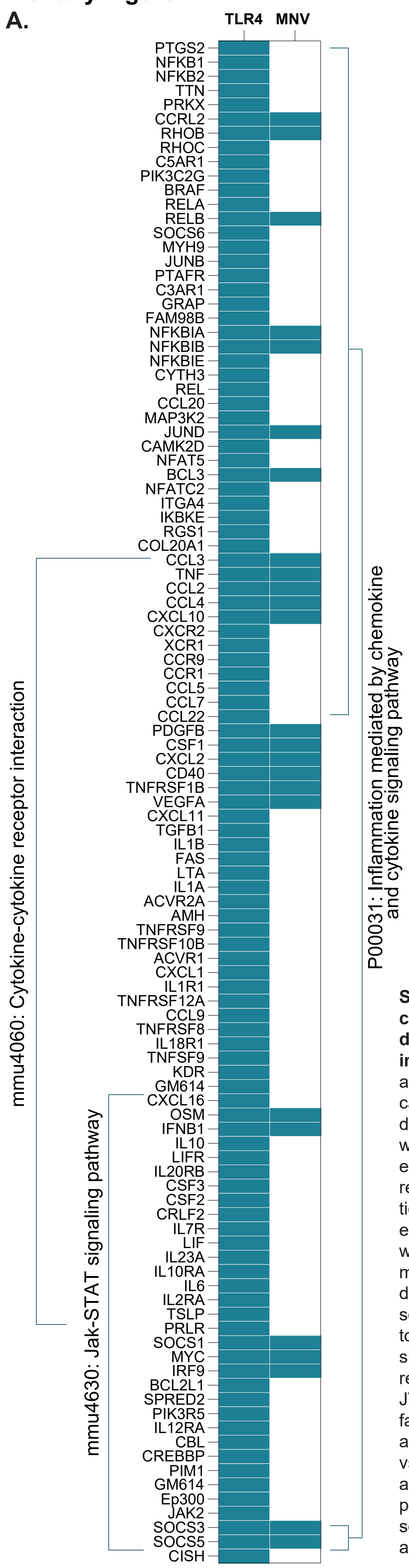

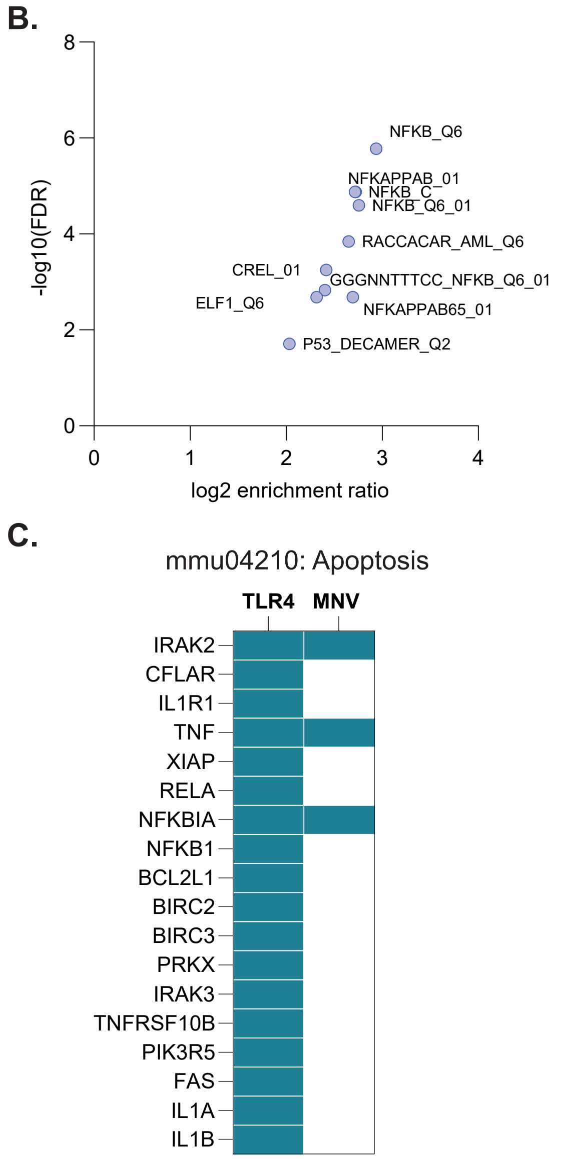

S4 Fig: Host response to MNV is lacking crucial pro-inflammatory cytokines, targets of JAK/STAT signalling and inflammation-related cell death factors linked to autocrine and paracrine activation of the innate immune response. (A) Comparison of the differentially expressed genes associated with the LPS activated TLR4 response in RAW264.7 cells with the significantly regulated genes in MNV-infected RAW264.7 cells at 10h p.i. Significantly differentially expressed gene, blue square, absence of differential expression, white square. List of cytokines had been generated from the list of differentially expressed genes (group 1) following LPS activation of RAW264.7 cells from (12) related to the pathway terms “mmu04060_Cytokine-cytokine receptor interaction”, “mmu04630_Jak-STAT signalling pathway”, “P00031_Inflammation mediated by chemokine and cytokine signalling pathway” generated using KEGG_pathways and Panther tools on the Cytoscape platform, gene symbols had been matched to fit the more recent annotation and run against the list of significant differentially expressed genes in MNV-infected cells at 10h p.i. using the open-source JVenn tool. (B) MNV-infected cells are lacking activation of genes related to NF-κB transcriptional network. Scatter plot of the transcriptional network analysis results for the “TLR4 only” genes regulated after TLR4 activation and not regulated in MNV-infected cells. “TLR4 only” list of genes generated from the JVenn comparison as in (A). Enrichments were calculated on the transcription factor target database MSigDB using the over-representation analysis method and plot created by biorthogonal projection of the log2 enrichment ration (x axis) vs the -log10(FDR) (y axis). (C) Comparison of the differentially expressed genes associated with the TLR4 response and enriched in “mmu04210_Apoptosis” pathway” KEGG pathway. Significantly differentially expressed gene, blue square, absence of differential expression, white square. List of genes generated as in (A).

Supplementary Figure 5

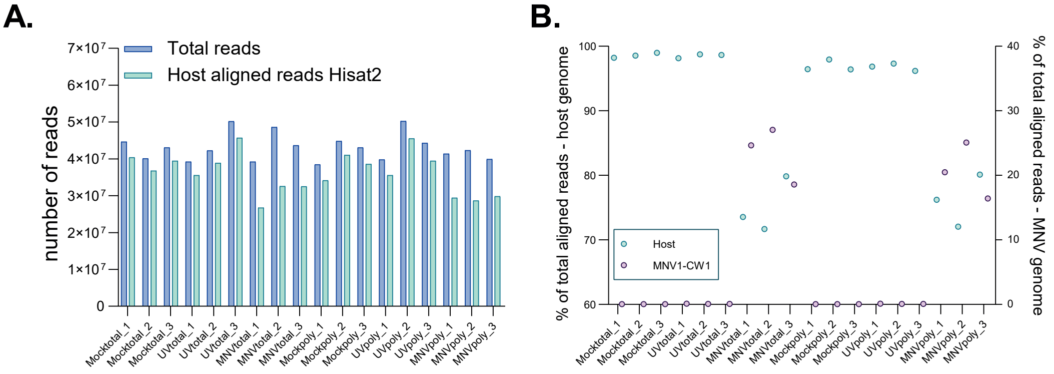

C.

	Hisat2 complete	Hisat2 filtered
all gene	17371	17195
protein coding	14711	14711
pseudogenes	483	483
ncRNA	1695	1647
snRNA	15	0
snoRNA	110	0
rRNA	3	0
other	12	12
unknown	342	342

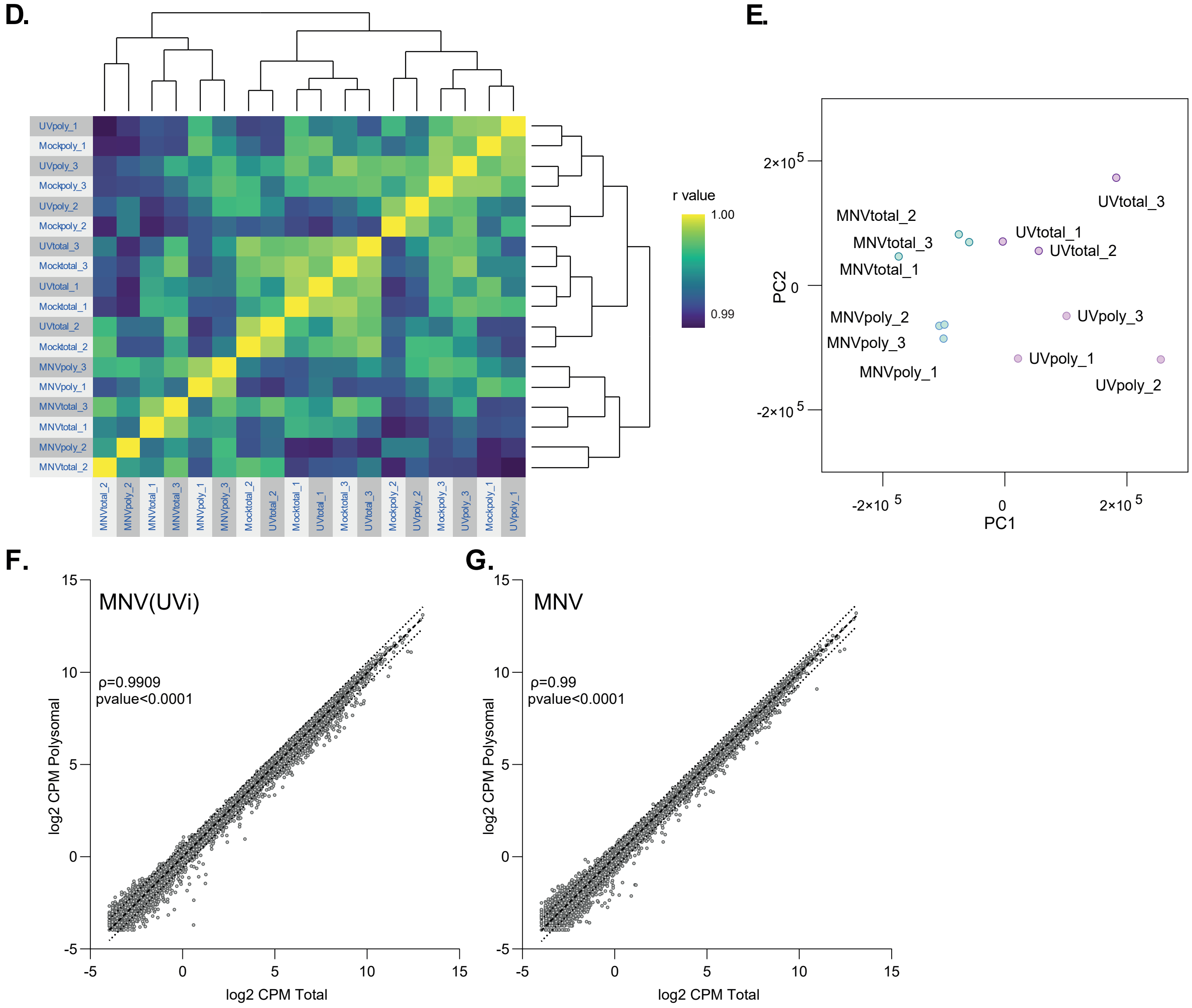

S5 Fig: Exploratory analysis of the RNA sequencing in BV2 cells. (A) Bar plot of the total number of reads for each sample (grey bars) and corresponding number of reads mapped to the host genome (GRCm38.p5 murine genome primary assembly) using Hisat2 (blue bars) software. (B) Scatter plot showing the percentage of total reads mapped to the murine genome using either Hisat2 (blue dots, left y axis) for each sample and the subsequent percentage of reads mapping to MNV1-CW1 genome (DQ285629) (purple dots, right y axis). (C) Mapped reads using Hisat2 were assigned a genomic feature (complete dataset) subsequently annotated using GenBank. All genes annotated as rRNA, miRNA, snoRNA, scaRNA were filtered out (filtered dataset). (D) Analysis of the batch effect of the biological replicates on the RNA sequencing outputs measured by calculation of the pair correlation of all samples from the complete Hisat2 (Host) and Bowtie (MNV) dataset, represented as a matrix. Correlation calculation using the Pearson method on log2CPM of the complete dataset filtered for low reads genes and small non-coding RNA, coupled with hierarchical clustering, showing the low impact of the batch effect with high correlation levels for samples of the same condition across the three replicates (r>0.98) and highlighting the clear sources of variation between the samples as "infection", and "fractions". (E) Representation of principal component analysis of the host dataset showing the "infection" and "fractions" as main sources of variability between the samples. Principal components scores were calculated on log2CPM datasets filtered for low reads genes and small non-coding RNA. (F–G) Scatter plots of the RNA sequencing results plotted as the average log2CPM in Polysomal (y axis) versus Total fraction (x axis) for MNV(UVi) (F) or MNV (G). The log2CPM of the host dataset genes were obtained by library size normalisation of the raw results and filtering out of the low reads genes. Correlation analysis showing the strong relationship (Spearman ρ>0.9, pval<0.0001) between the Total and Polysomal fractions. Dotted lines, linear regression and 95% confidence intervals.

Supplementary Figure 6

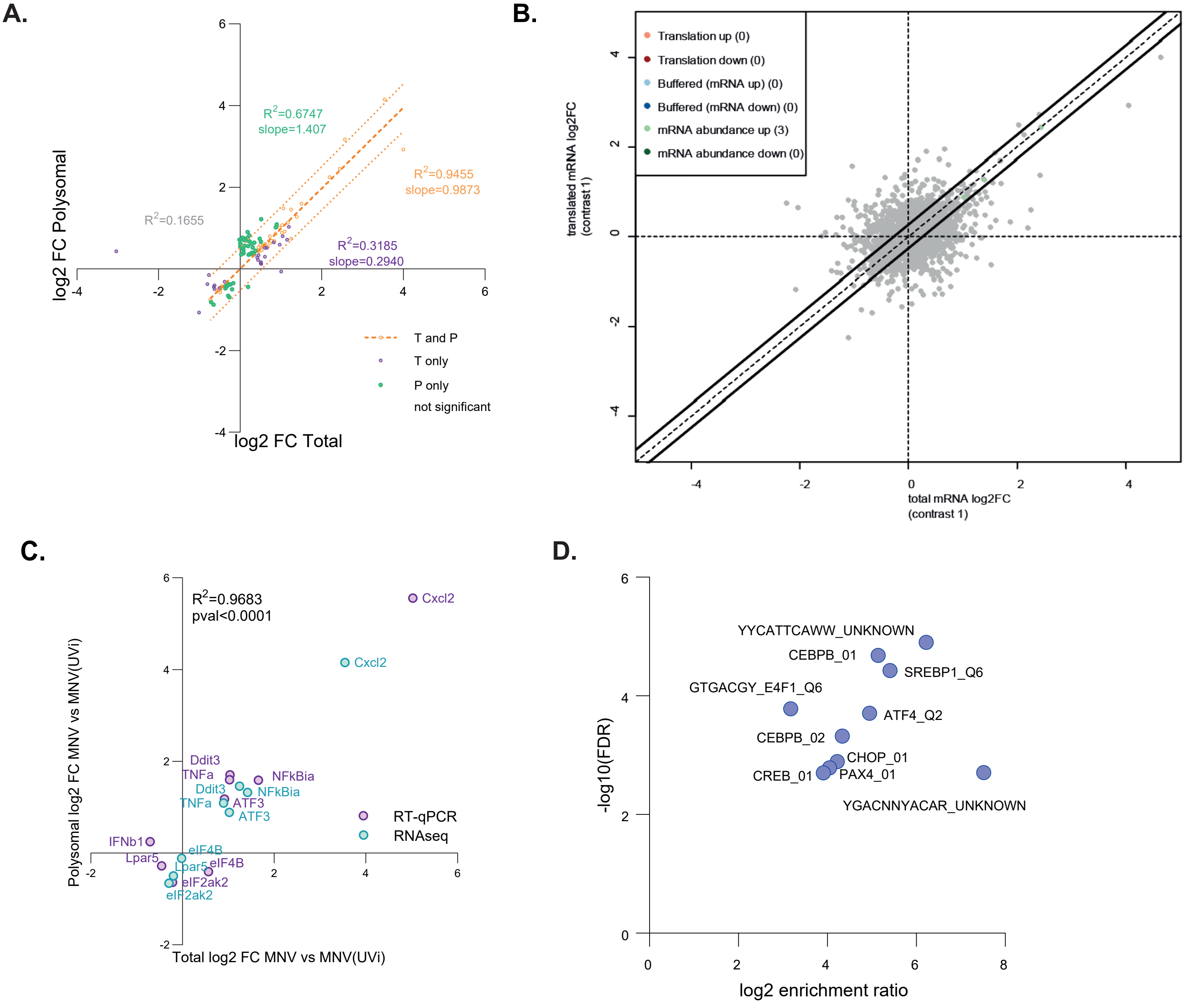

S6 Fig: Differential expression analysis of MNV infection in BV2 cells. Differential analysis performed on RNA sequencing data showed a genetic reprogramming induced by MNV replication in BV2 cells lacking IFN, NF-κB and Jak/Stat response. (A) Scatter plot of the results of the differential expression analysis comparing MNV-infected cells versus MNV(UVi)-infected cells at 10 hpi in the Total (x axis) versus the Polysomal fraction (y axis) showing mainly a subset of MNV-specific upregulated genes and few downregulated genes with similar behaviour in both fractions. The differential expression analysis was performed on all samples. using the multivariate tool GLMEdgeR and gene level changes were considered significant when associated with a BHpval <0.1. Results expressed as log2 (FC MNV vs MNV(UVi)) of the complete gene dataset were used for the biorthogonal projection and subdivided into four groups: genes significant in both Total and Polysomal fractions (orange dots), genes significant in Total fraction only (purple dots), genes significant in Polysomal fraction only (green dots), and genes displaying no changes (grey dots). Correlation analysis using the Pearson method showed high degree of correlation for the three significant groups as indicated on the plot. Dotted orange lines, linear regression and prediction band at 95% of confidence for the “significant in both fractions” genes. Most significant genes fall into this prediction of significance in both fractions reflecting the stringency of the significance cut-off and the likeliness of changes in both fractions for all the significant genes. (B) Scatter plot of the translational control analysis results showing an absence of translational regulation in MNV-infected cells, translated mRNA (y axis) versus Total mRNA (x axis). (C) Validation by qRT-PCR of the translatomic results. Scatter plot of the log2FC from the differential expression analysis (green dots) for the contrasts MNV-infected cells versus MNV(UVi)-infected cells in Total (x axis) versus Polysomal (y axis) fraction compared with the log2FC obtained by qRT-PCR (purple dots) showing a high degree of correlation (Pearson correlation coefficient r = 0.9840, pval<0.0001). (D) Scatter plot of the transcriptional network analysis results for the MNV-induced upregulated genes at 10 hpi showing the absence of IRF activation and prevalence of ATF4 and CHOP regulatory networks. Enrichments were calculated on the transcription factor target database MSigDB using the over-representation analysis method and plot created by biorthogonal projection of the log2 enrichment ratio (x axis) versus the corresponding -log10(FDR) (y axis).

Supplementary Figure 7

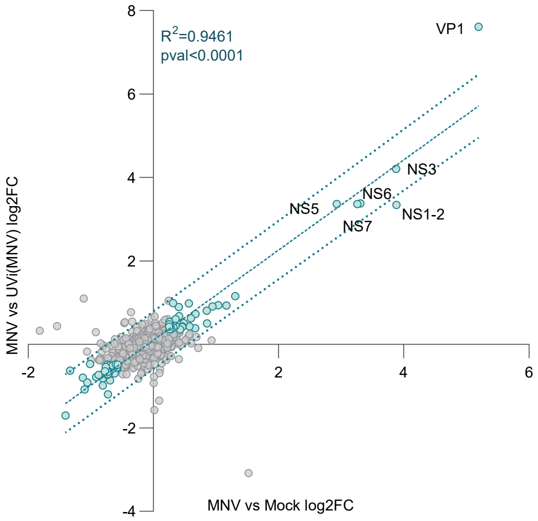

S7 Fig.: Neotranslatome analysis at 6h p.i. RAW264.7 cells proteins were subjected to labelling with media supplemented with Light (R0K0), Medium (R6K4) or Heavy (R10K8) Arginine and Lysine for at least 5 passages before assessment of their suitability to the proteomics analysis. Biorthogonal projection of the proteomics analysis results plotting the genes significant (blue dots) in both contrasts for cells mock-, MNV(UVi), or MNV-infected at 6h p.i., linear regression analysis showing a high level of correlation (Pearson r =0.9727, pval<0.0001).

Supplementary Figure 8

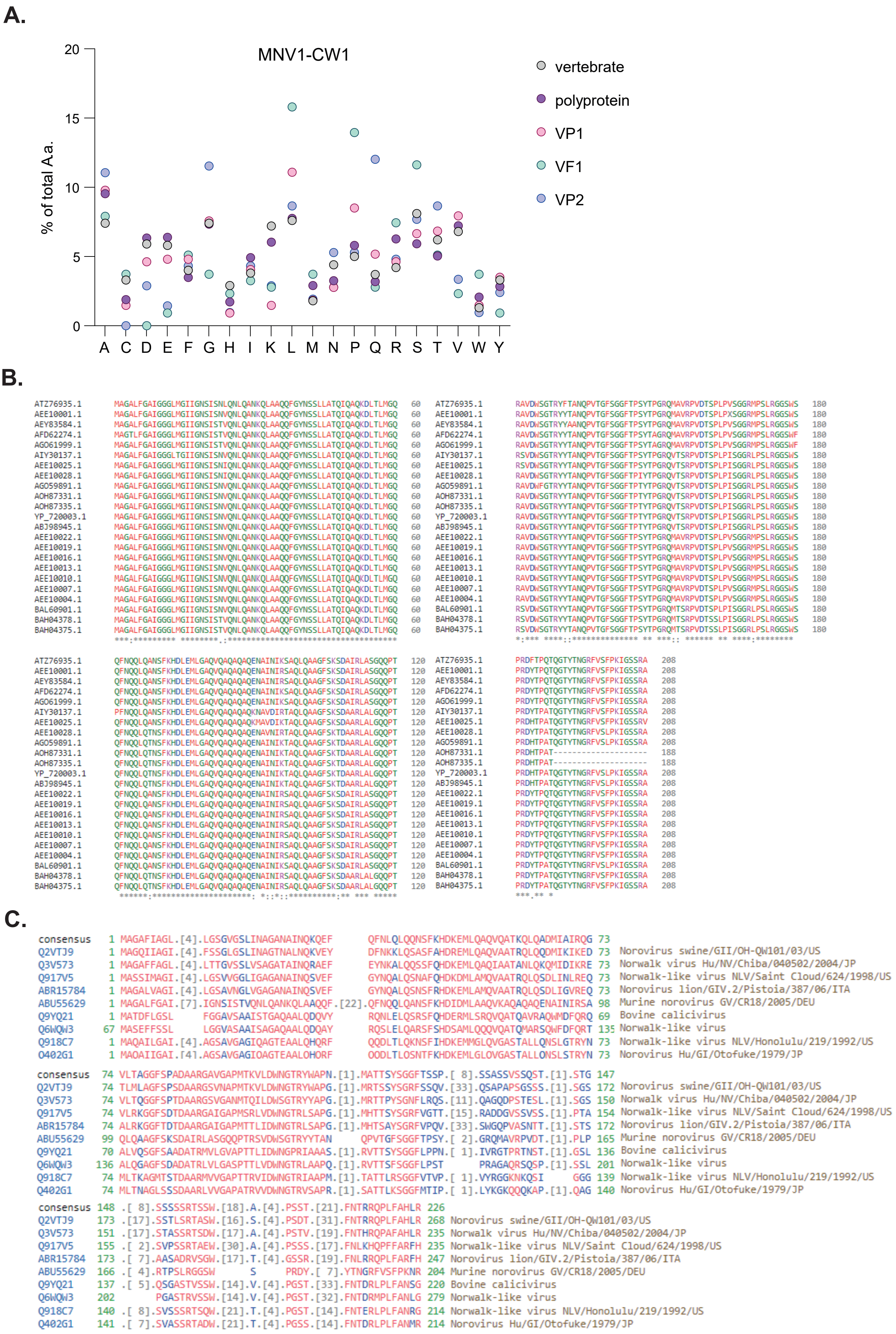

S8 Fig: Murine Norovirus small basic protein VP2 is enriched in glutamine residues. (A) Analysis of the amino usage in MNV1 CW1 proteins (polyprotein YP_720001.1 purple, VP1 YP_720002.1 pink, VP2 YP_720003.1 blue, VF1 YP_006390081.1 green) showed enrichment of VP2 in glutamine compared to the average usage in vertebrate (grey) and other MNV1 proteins (B). Conservation analysis between murine norovirus strains showing a very high level of conservation of VP2. (C) Protein superfamily analysis using the Conserved Protein Domain Family tool in NCBI on pfam 03035 (RNA_capsid) showing a partial conservation of the glutamine usage in VP2 of other Caliciviridae family members.
